# Impact of chemorophylaxis policy for AIDS-immunocompromised patients on emergence of bacterial resistance

**DOI:** 10.1371/journal.pone.0225861

**Published:** 2020-01-30

**Authors:** Ashley A. DeNegre, Kellen Myers, Nina H. Fefferman

**Affiliations:** 1 Department of Ecology, Evolution and Natural Resources, Rutgers University, New Brunswick, New Jersey, United States of America; 2 The Command, Control and Interoperability Center for Advanced Data Analysis (CCICADA), Rutgers University, New Brunswick, New Jersey, United States of America; 3 Department of Ecology & Evolutionary Biology, University of Tennessee, Knoxville, Tennessee, United States of America; 4 Department of Mathematics, University of Tennessee, Knoxville, Tennessee, United States of America; 5 National Institute for Mathematical and Biological Synthesis (NIMBioS), University of Tennessee, Knoxville, Tennessee, United States of America; 6 Department of Mathematics, Tusculum University, Greeneville, Tennessee, United States of America; Universidad Nacional de la Plata, ARGENTINA

## Abstract

Chemoprophylaxis (antibiotic prophylaxis) is a long relied-upon means of opportunistic infection management among HIV/AIDS patients, but its use represents an evolutionary tradeoff: Despite the benefits of chemoprophylaxis, widespread use of antibiotics creates a selective advantage for drug-resistant bacterial strains. Especially in the developing world, with combined resource limitations, antibiotic misuse, and often-poor infection control, the emergence of antibiotic resistance may pose a critical health risk. Extending previous work that demonstrated that this risk is heightened when a significant proportion of the population is HIV/AIDS-immunocompromised, we work to address the relationship between HIV/AIDS patients’ use of antibiotic chemoprophylaxis and the emergence of resistance. We apply an SEIR compartmental model, parameterized to reflect varying percentages of chemoprophylaxis use among HIV/AIDS+ patients in a resource-limited setting, to investigate the magnitude of the risk of prophylaxis-associated emergence versus the individual-level benefits it is presumed to provide. The results from this model suggest that, while still providing tangible benefits to the individual, chemoprophylaxis is associated with negligible decreases in population-wide morbidity and mortality from bacterial infection, and may also fail to provide assumed efficacy in reduction of TB prevalence.

## Introduction

The use of antibiotic prophylaxis has long been relied upon to reduce morbidity and mortality due to opportunistic infection among the HIV/AIDS-immunosuppressed–especially when access to antiretrovirals is limited [1–6]. However, the use of chemoprophylaxis (e.g. antibiotic prophylaxis) among the HIV/AIDS-immunocompromised patients represents an evolutionary tradeoff, wherein the success of chemoprophylaxis must be weighed against its potential to contribute to antimicrobial resistance by selecting for drug-resistant bacterial strains.

The rapidity with which antibiotic resistance can develop represents a worldwide threat to infection prevention and treatment, manifesting on the scale of months or even weeks [7–10]. The risk of emergence is magnified in the developing world, where resource constraints limit both infection management and resistance monitoring, and where antimicrobial misuse is rampant due to factors such as inconsistent availability and poor drug quality [11–14]. Moreover, HIV/AIDS incidence and prevalence, worldwide, are highest within developing nations [15–18]. We recently demonstrated that, in such HIV/AIDS-prevalent settings, the disrupted selective pressures associated with widespread population-level immunoincompetence (as compared to those usually applied by the combination of antibiotic therapy and immune activation [19]) further contribute to the emergence of antibiotic-resistant bacterial infections [20]. Having chosen Swaziland and Indonesia–which, respectively, represent the upper and lower bounds of HIV/AIDS prevalence within the developing world in 2013 [21, 22]–as sample populations, we now examine the effects of chemoprophylaxis use by the HIV/AIDS-immunocompromised on the probability of emergence.

Our previous study relied on the simplifying assumption that antibiotic use referred specifically to targeted antimicrobials, since the high prevalence of bacterial infections that is characteristic of the developing world necessitates frequent use of the use of curative antibiotics [23–26]. However, as exemplified by pathogens such as *Mycobacterium tuberculosis* and *Staphylococcus aureus*, the incidence of bacterial infections, despite the use of broad-spectrum chemoprophylaxis to combat them, is indicative of the emergence of resistance [4, 27–30]. (While it is outside the scope of this paper, we note that chemoprophylaxis prescriptions for the HIV/AIDS-immunocompromised also include antiviral and antiparasitic agents, which can select for resistant viral and parasite strains, respectively [3, 31–33].) Given that total, population-wide, emergence is the sum of the resistant infections attributable to curative antibiotic use and those arising due to the use of chemoprophylaxis, we now analyze the extent to which chemoprophylaxis affects the emergence of drug-resistant bacterial strains.

Even when antibiotics are used correctly, widespread antimicrobial use for the treatment of active infections selects for the emergence of resistance [14, 26]. Based on data specific to curative antibiotic use among both immunocompetent and HIV/AIDS+ hosts, we suspect that many factors, including financial constraints, sociocultural perspectives on medicine, patients’ mental and physical health and drug regulation [11, 12, 34–37], could lead to considerable variation in the use and availability of chemoprophylaxis. However, among the HIV/AIDS-immunocompromised, an additional economic barrier exists: Although HIV/AIDS prevalence varies greatly between Indonesia (0.46%) and Swaziland (27.4%) [21, 22], the widespread poverty common to both regions suggests that those with HIV/AIDS may, at some point, need to choose between purchasing chemoprophylaxis and purchasing highly active antiretroviral treatment (HAART) [38, 39]. This constraint could lead to variability in the proportion of HIV/AIDS+ susceptibles actively using chemoprophylaxis at any given time, thereby also increasing variation in projected prophylaxis-attributable emergence.

There is ample evidence that misuse of curative antibiotics, which includes overuse, self-medication, and inconsistent dosing, is the greatest predictor of emergence [26, 40–49]. However, the degree to which the use of chemoprophylaxis within HIV/AIDS-prevalent regions contributes to local and worldwide emergence is not readily available in the literature. Despite the potential health benefits of chemoprophylaxis to HIV/AIDS+ susceptibles, we cannot ignore the possibility that the risk of heightened emergence in HIV/AIDS-prevalent populations [20] may be compounded by an increase in selection for resistant bacteria arising out of chemoprophylaxis use. Drug-resistant infections are particularly problematic among HIV/AIDS-immunocompromised hosts, given their increased susceptibility to opportunistic pathogens, as well as the accelerated progression from HIV to active AIDS that occurs as a result of chronic, infection-mediated, immune activation [50–52]. Therefore, in an effort to promote antibiotic-prescribing decisions that minimize resistance arising out of the use of chemoprophylaxis, we present an SEIR compartmental model to quantify the relative contribution to total emergence that results from chemoprophylaxis use in HIV/AIDS patients, given the many factors that may limit its availability in the developing world. We also analyze the risk-benefit relationship between prophylaxis-associated emergence, and the health advantages chemoprophylaxis use is presumed to offer the HIV/AIDS-immunocompromised, by examining the factor change in morbidity and mortality among HIV/AIDS+ susceptibles as chemoprophylaxis use is varied.

## Materials & methods

Holding HIV/AIDS prevalence constant at 0.46% and 27.4% for Indonesia and Swaziland [21, 22], respectively, we address the question of how use of chemoprophylaxis contributes to the emergence of antibiotic resistance. In recognition of the need for curative antibiotics among the fully immunocompetent, and those with HIV/AIDS who are not prescribed chemoprophylaxis, we include a parameter representing the probability of antibiotic adherence among those with active infections. We assume that HIV/AIDS+ infectives receiving targeted antibiotics are not simultaneously being treated with broad-spectrum chemoprophylaxis.

Given the time-scale of interest for bacterial infections, we do not focus on host HIV/AIDS status as an epidemiological process. We note that susceptibles are categorized based on immune status as follows: those who are fully immunocompetent (i.e., HIV/AIDS-negative); those whose immune function is compromised by active AIDS; and those who are HIV or AIDS+, but whose consistent use of HAART enhances their immunocompetence such that their risk of succumbing to complications of AIDS-defining illness is low [53–55]. The rapid nature of bacterial evolution allows us to observe emergence of resistance over a relatively short time period–in this case 180 days–during which we assume HIV/AIDS status remains constant, with no seroconversion occurring [53].

Because we also describe hosts in terms of bacterial infection status and adherence to targeted antibiotics, we attach super- and subscripts to the variables associated with each compartment of the model. In keeping with previous work [20], we use a ^superscript^ to dually describe HIV and HAART status; and we use a _subscript_ to describe both bacterial infection status and adherence to antibiotics. In addition to the previously defined notational designations, we also adopt the subscript combination “+-” to describe infection-negative, prophylaxis-positive, susceptibles, thereby distinguishing them from hosts using antibiotics to treat active infection (all possible super- and subscript combinations appear in Table A in S1 Appendix).

We also include the composite probability of emergence of and success of an antibiotic resistant infection among prophylactically-treated susceptibles (ϕ). Values for ϕ (S1 Appendix) vary based on immune status and antibiotic adherence, and were determined based on: a combination of the per-cell, per-bacterial generation mutation rates; the total number of infected cells per host; the expected number of bacterial generations per infection duration; the per-category infection duration; and the relative success of the mutant strain [56–59]. For the purposes of this model, antibiotic resistance is defined functionally, meaning specifically that the pathogen replicates, disease propagates, and transmission may occur despite antibiotic treatment.

We make two conservative assumptions: First, we assume that those susceptibles prescribed chemoprophylaxis are completely adherent to their dosing instructions. Second, we assume that those who develop resistant infections while using chemoprophylaxis are then also completely adherent to the targeted antibiotics used to treat the active infection. (As such, all infectives who have previously been treated prophylactically are assigned to either the $I_{++}^{+-}$ or $I_{++}^{++}$ categories, depending on HIV/HAART status.) Consistent with these assumptions, we assign $\phi_{-+}^{+-}$ and $\phi_{-+}^{++}$ values equivalent to those assigned to fully antibiotic adherent AIDS+/HAART- and HIV/AIDS+/HAART+ hosts, respectively.

**Fig 1:**
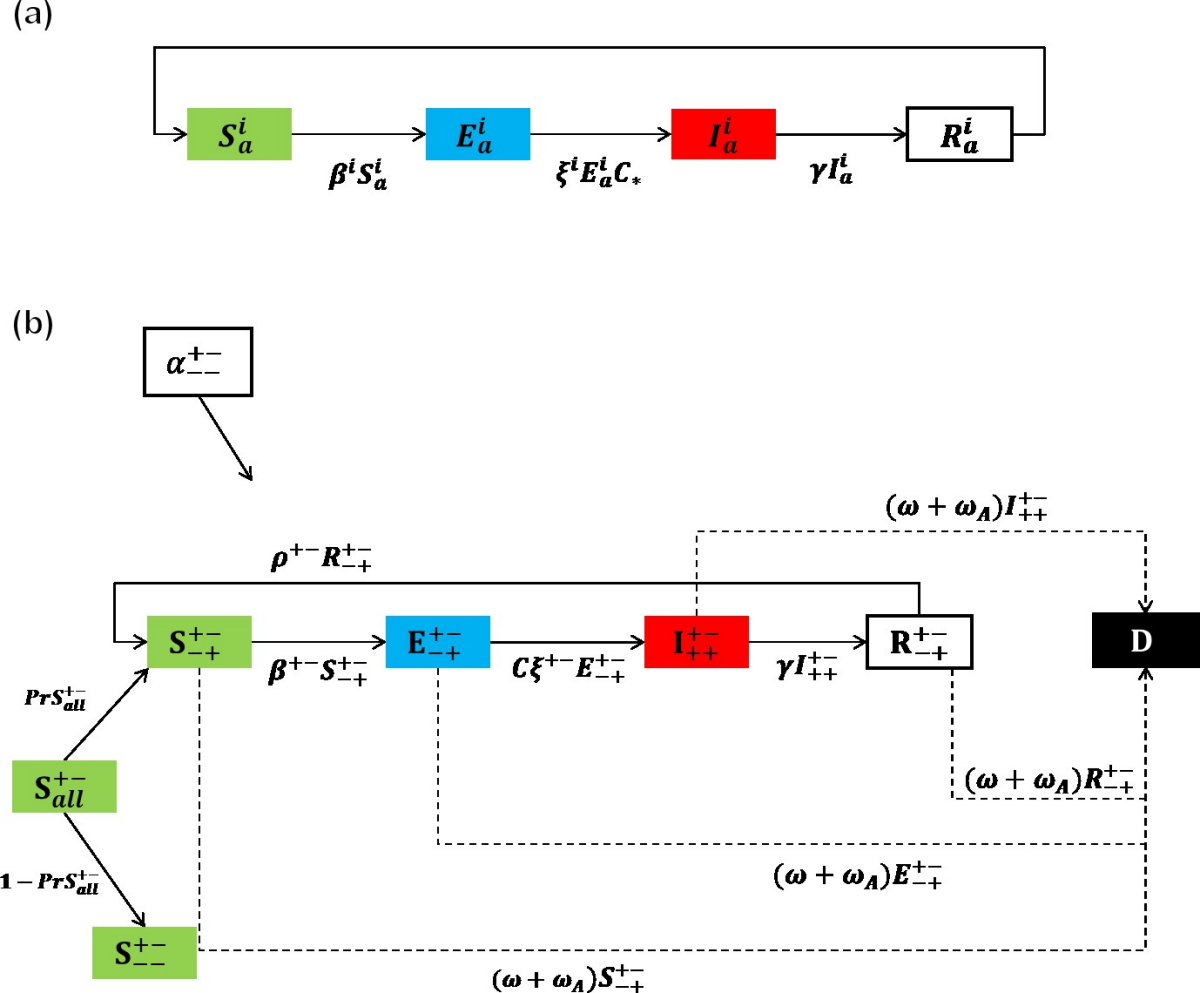
SEIR transmission dynamics. (a) A basic SEIR model (assuming a closed system), wherein health status changes from susceptible to exposed at a rate of β, from exposed to infectious at a rate of ζ, and infectious to recovered at a rate of γ. The super- and subscripts “i” and “a" are used generically to demonstrate that there are many possible host health outcomes, depending on the combination of immune status and antibiotic-taking behavior. (b) Flowchart depicting transmission dynamics specific to our model; we note all possible progressions for a prophylactically-treated HIV/AIDS+, HAART- host.

Using tuberculosis as an example pathogen, the model follows the progression of TB infection throughout a population separated by immune status (Fig 1) and measures the combined impact of prophylactic and targeted antibiotic treatment (with different levels of adherence among the actively infective) on the emergence of antibiotic resistance. The entire system is described by a set of ordinary differential equations (S1 Appendix), which also captures TB-specific mortality for all infectives. We assume zero mortality among fully immunocompetent and HAART+ infectives when antibiotic protocols are followed completely. (Table E in S1 Appendix contains a detailed list of parameters introduced in this model, including their condition dependencies, values used, and the reference(s) from which they were estimated; and S1 and S2 Appendices detail the methods by which parameter values were calculated).

The significant difference in HIV/AIDS prevalence between Indonesia (0.46%) [21] and Swaziland (27.4%) [22] suggests that the proportion of HIV/AIDS+ susceptibles–regardless of HAART status–using chemoprophylaxis at any given time is likely to vary considerably between the two countries. This ensures their suitability as extremal comparators as we examine the impact of chemoprophylaxis use on the emergence of antibiotic resistance.

In surveying a subset of HIV+ Italians, Napoli, et al. [60] found that 40% had been treated prophylactically against *Pneumocystis carinii*. However, since this refers to a specific patient population (HIV+ susceptibles not yet considered AIDS-immunocompromised) from the developed world, we suspect that it is unlikely to be representative of treatment percentages within the developing world. We therefore investigated the impact on emergence of varying the percentage of HIV/AIDS+ susceptibles being treated prophylactically in 10% increments from 0–100%, with the trial in which chemoprophylaxis is not used acting as a negative control. In the absence of any data regarding the prevalence of chemoprophylaxis use in the developing world, we based this range on reported adherence to targeted antibiotics. Adherence varies based on factors such as age, socioeconomic status and the drug’s side-effect profile [61–63]. Varying this parameter from 0% to 100% allows us to explore the full range of possible adherence rates, for which we have no specific data, while still being used in composition with parameters that have been empirically determined. We expect that similar factors will influence the use and availability of chemoprophylaxis, thereby also influencing the likelihood of treatment.

### Chemoprophylaxis-specific emergence

When infections occur in HIV/AIDS+ susceptibles despite the intended effects of chemoprophylaxis, they likely arise out of drug-resistant mutations within the host. Within this initial exploration, we make the simplifying assumption that resistant strains do not circulate within the population, though follow-up efforts will explore competition between sensitive and resistant strains.

### Probability of emergence

To quantify the relative contributions of both broad-spectrum chemoprophylaxis and targeted antibiotics to the emergence of resistance, we established a composite probability of the emergence of resistance for each possible combination of treatment and immune status. The values for this parameter are informed by the TB literature, and are designated as $\phi_{rx}^{i}$, where “i” and “rx” are used generically to designated immune and treatment status, respectively. (We note that $\phi_{rx}^{i}$ is calculated from the results of the ODE model; see S1 Appendix). We multiply category-specific $\phi_{rx}^{i}$ values by the total number of infectives corresponding to each category to determine the expected number of resistant infections as percent chemoprophylaxis treatment is varied. Finally, we calculate the prophylaxis-dependent relative emergence specifically attributable to each category of infective.

## Results

### Chemoprophylaxis-dependent emergence

**Fig 2:**
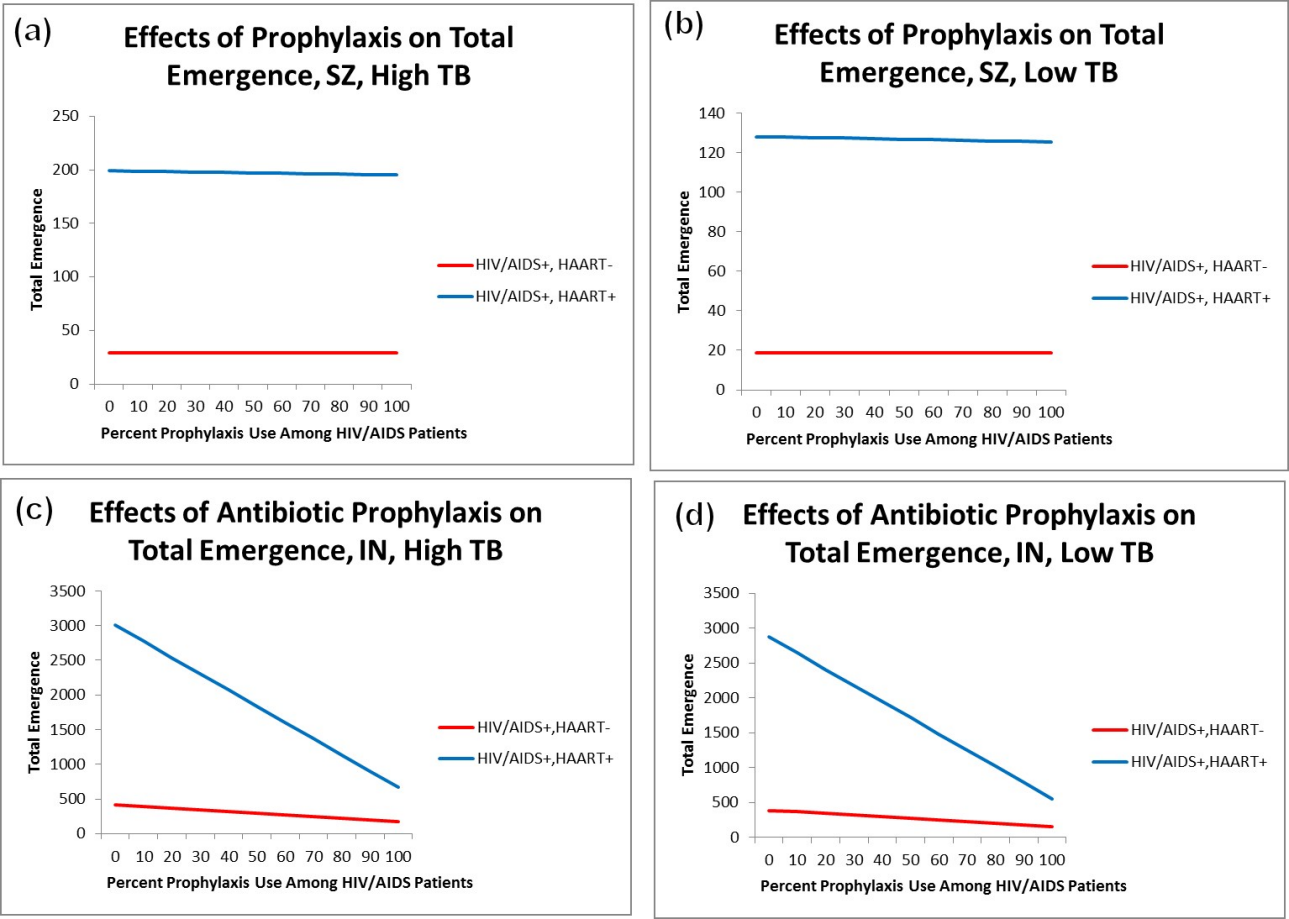
Effect of chemoprophylaxis on expected emergence. The impact of chemoprophylaxis on emergence of resistance varied considerably between Swaziland (a-b) and Indonesia (c-d). While chemoprophylaxis use does not actively contribute to the emergence of resistance in Swaziland, its neutral effect on emergence suggests that chemoprophylaxis may not be managing TB incidence effectively. Conversely, use of chemoprophylaxis in Indonesia dramatically reduces the incidence of drug-resistant TB in HIV/AIDS patients.

When we compared the expected emergence of antibiotic-resistant TB in Swaziland to that in Indonesia, we observed a drastic difference in the impact of chemoprophylaxis. In Swaziland, irrespective of initial TB prevalence, there was a negligible difference in expected emergence among actively immunocompromised (HIV/AIDS+, HAART-) HAART-treated patients (Fig 2). Given that we assumed no resistant strain circulation occurs, we would not expect chemoprophylaxis to have a significant effect on the number of drug-resistant infections arising among the fully immunocompetent since they were not provided chemoprophylaxis. However, even when 100% of HIV/AIDS+, HAART- susceptibles received chemoprophylaxis, there was a less than 1% decrease in the emergence of resistance (Table 1 and Fig 2). The greatest observed impact occurred among HIV/HAART+ hosts, with a 2% reduction in emerging resistance when 100% chemoprophylaxis use was assumed. This suggests that chemoprophylaxis cannot be relied upon as a means of emergence control in Swaziland.

**Table 1:** Factor changes in emergence, Swaziland. Using 0% chemoprophylaxis treatment as a baseline, we calculated the factor differences in cases of emerging antibiotic-resistant TB, occurring in HIV/AIDS+ hosts, as percent chemoprophylaxis use was varied in 10% increments. Given that increasing chemoprophylaxis use had a negligible effect on the emergence of resistance in Swaziland, we have included only the 0, 50 and 100% chemoprophylaxis conditions for purposes of illustration. We note that we use “*” to represent all possible TB/curative antibiotic combinations that can occur with a particular immune status.

Population	TB Prevalence	Immune Status	Percent Chemoprophylaxis Use (HIV/AIDS+)	Expected Emergence (Total Cases)	Factor Difference in Emergence
Swaziland	Low	$I_{*}^{+-}$	0%	18.68	-
			50%	18.65	0.99
			100%	18.62	0.99
		$I_{*}^{++}$	0%	128.07	-
			50%	126.74	0.99
			100%	125.40	0.98
	High	$I_{*}^{+-}$	0%	29.07	-
			50%	29.03	0.99
			100%	28.99	0.99
		$I_{*}^{++}$	0%	199.2	-
			50%	197.2	0.99
			100%	195.17	0.98

**Fig 3:**
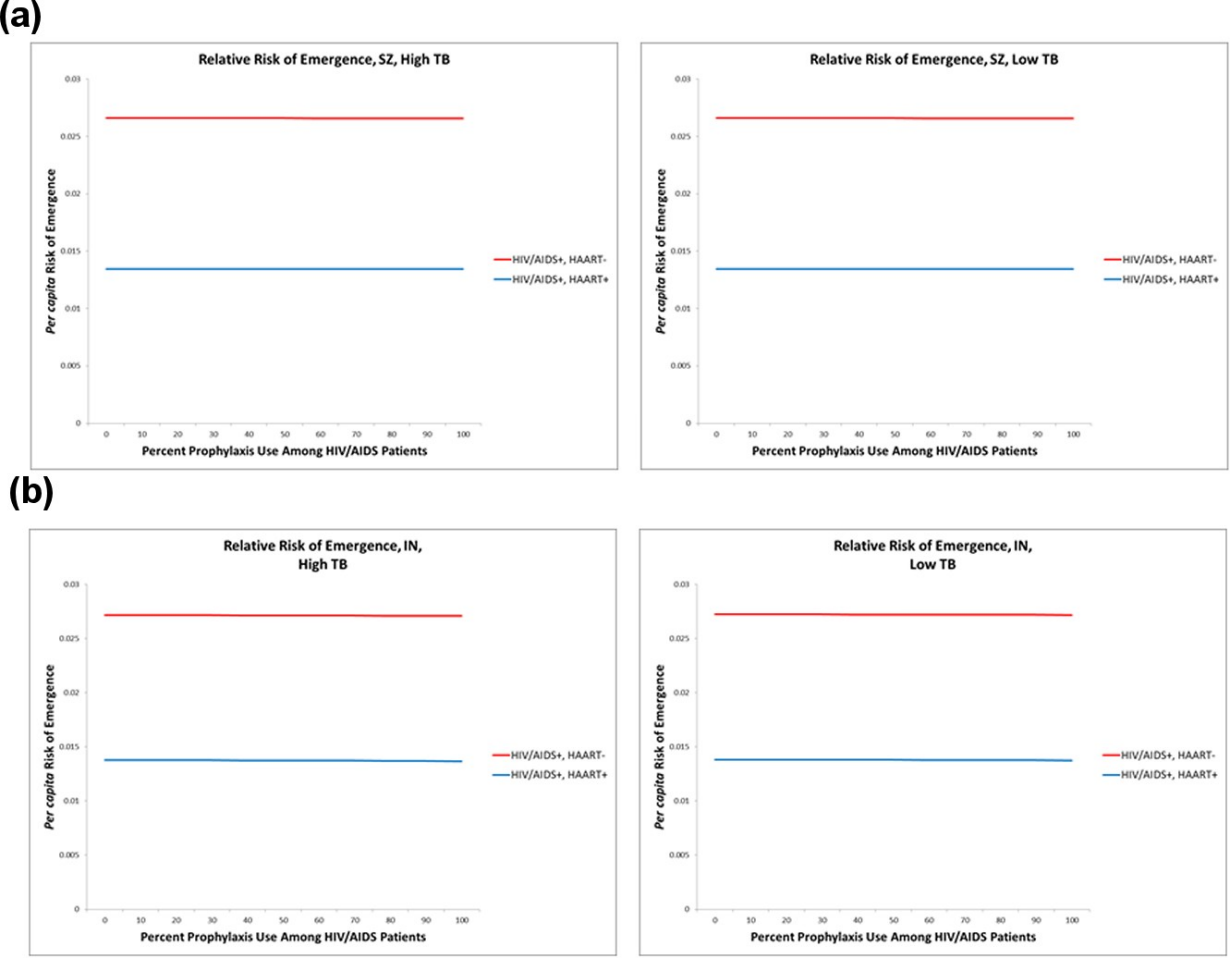
Expected per capita emergence. When we examined the per capita risk of emergence associated with both HAART+ and HAART- patients, we found that drug-resistant TB infections are twice as likely to arise in those who do not receive HAART (i.e., those with active AIDS). We observed this result in both Indonesia and Swaziland, irrespective of both initial TB prevalence and percent chemoprophylaxis treatment. The asterisk * denotes any possible subscript, as these parameters do not vary by subscript.

Contrary to the results from Swaziland, we note a sharp decline in risk of prophylaxis-associated emergence in Indonesia, where, among the actively AIDS-immunocompromised, we observed a nearly 60% reduction in risk when 100% of HIV/AIDS+ susceptibles are prophylactically treated. Among those who are HIV/AIDS+ and HAART-adherent, the impact was even greater; risk of emergence was reduced by approximately 80%. These results occurred irrespective of initial TB prevalence (Table 2 and Fig 2). Despite this result, when we analyzed per capita risk of emergence in HIV/AIDS patients, we found that in both Indonesia and Swaziland, drug-resistant TB infections are still approximately twice as likely to emerge among those with active AIDS as among those receiving HAART (Fig 3). This suggests that increased HAART access may be a valuable means of managing TB in HIV+ patients.

**Table 2:** Factor changes in emergence, Indonesia. The impact of chemoprophylaxis on emergence in Indonesia varied considerably, relative to Swaziland. Again using 0% chemoprophylaxis treatment as a baseline, we found that risk of emergence could be reduced by approximately 60% among HIV+/AIDS+, HAART-infectives, and by approximately 80% among HIV/AIDS+, HAART+ infectives. We note that we use “*” to represent all possible TB/curative antibiotic combinations that can occur with a particular immune status.

Population	TB Prevalence	Immune Status	Percent Chemoprophylaxis Use (HIV/AIDS+)	Expected Emergence (Total Cases)	Factor Difference in Emergence
Indonesia	Low	$I_{*}^{+-}$	0%	390.17	-
			50%	273.41	0.70
			100%	156.72	0.40
		$I_{*}^{++}$	0%	2878.52	-
			50%	1717.98	0.60
			100%	557.62	0.19
	High	$I_{*}^{+-}$	0%	412.43	-
			50%	293.28	0.71
			100%	174.22	0.42
		$I_{*}^{++}$	0%	3006.82	-
			50%	1856.37	0.61
			100%	667.105	0.22

Recall that in both Indonesia and Swaziland we have made the conservative assumptions that prophylactically-treated susceptibles adhere completely to dosing instructions, and that they continue to do so once they become infected with a bacterial pathogen resistant to broad-spectrum chemoprophylaxis and must change antibiotics to treat the infection. We may therefore be conservatively underestimating total emergence, but we do not expect that this will qualitatively impact the results of our model.

### Influence of chemoprophylaxis on infectivity and mortality

Using expected TB incidence under conditions of 0% chemoprophylaxis use as a baseline, we analyzed the factor change in population-wide infectivity that results from varying the percentage of HIV/AIDS+ susceptibles using chemoprophylaxis. While we did observe a decline in population-wide TB incidence Swaziland, as chemoprophylaxis use was increase from 0–100%, it was not as pronounced as we might have expected, given the reliance upon chemoprophylaxis as a means of minimizing opportunistic infection risk among HIV/AIDS patients [64]. Under conditions of both low and high TB prevalence, we observed the greatest decline in infectivity among HIV/AIDS+, HAART+ individuals. However, incidence was reduced by only 2%, relative to the baseline, when 100% of HIV/AIDS+ susceptibles were prescribed chemoprophylaxis.

We note that, when 100% of HIV/AIDS+ susceptibles are treated prophylactically, 27.4% of the population should be protected [22]. We would, therefore, expect a proportionate decrease in TB prevalence; however, this was not the case. We instead found that the benefits of chemoprophylaxis are overwhelmed by the risk attributable to transmission by current infectives. Unfortunately, this mitigates the intended impact of chemoprophylaxis.

Our mortality results mirrored those from the analysis of TB incidence in Swaziland. Again, despite a goal of chemoprophylaxis being to reduce bacterial infection-related mortality among the HIV/AIDS-immunocompromised, the decline in mortality as percent chemoprophylaxis treatment increased was nearly undetectable (<1%), irrespective of immune status or initial TB prevalence.

Consistent with our findings regarding emergence in Indonesia, we also observed a more noticeable effect of chemoprophylaxis when we examined TB-incidence and TB-associated mortality among HIV/AIDS-affected individuals. As chemoprophylaxis use among HIV/AIDS+ susceptibles increased from 0–100%, we observed a corresponding decline in TB incidence. Relative to the baseline, TB incidence among HIV/AIDS+, HAART- hosts is reduced by 60% when initial TB prevalence is low, and by 58%, when initial prevalence is high. TB-attributable mortality among HIV/AIDS+, HAART+ hosts is reduced by 81% in the low TB condition, and by 78% in the high TB condition.

**Fig 4:**
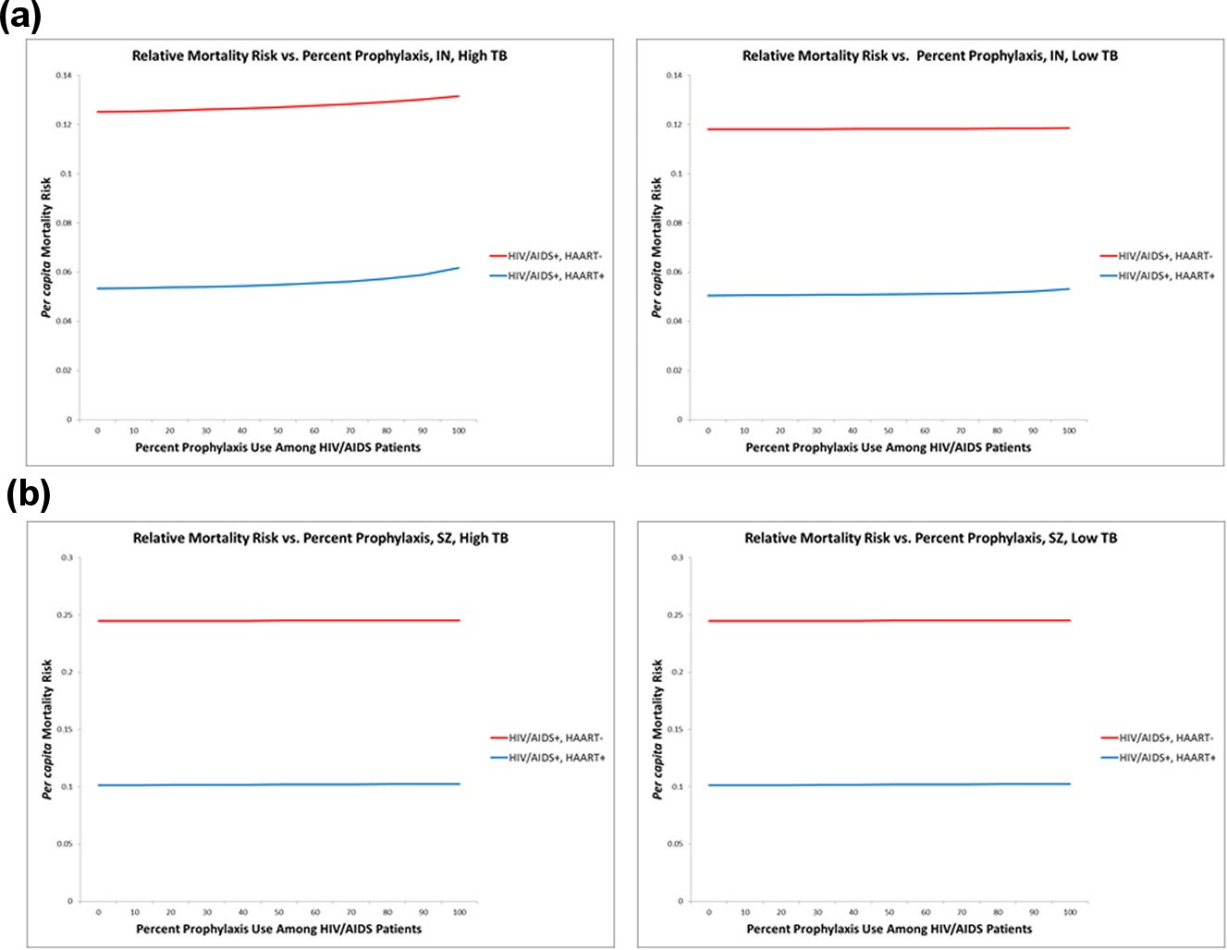
Expected per capita TB-associated mortality. Our analysis indicates that HIV/AIDS patients not receiving HAART are, individually, twice as at risk of TB-related mortality as those who are HAART treated. This result occurred in both Indonesia and Swaziland, regardless of both initial TB prevalence and the proportion of HIV/AIDS patients receiving chemoprophylaxis.

As would be expected given the decline in TB incidence, TB-associated mortality also decreased with increased chemoprophylaxis treatment. When initial TB prevalence was low, and 100% of HIV/AIDS+ susceptibles were prophylactically treated, HIV/AIDS+, HAART- hosts benefitted from a 60% reduction in mortality; and mortality in HIV/HAART+ hosts declined by 80%. Under the high TB condition, mortality was reduced by 55% among the actively AIDS-immunocompromised, and by 74% among those receiving HAART. We note, however, that our analysis of per capita mortality among HIV/AIDS patients demonstrated that those with active AIDS experience twice the risk of TB-associated mortality as those who are HAART-treated (Fig 4).

### Sensitivity analysis

We performed a sensitivity analysis to determine which parameters had the greatest impact on the disease burden among each of the three populations I^––^, I^+–^, and I^+ +^. In each of the eight possible combinations of Swaziland vs Indonesia, high TB prevalence vs low, and with or without AIDS, we compiled 100 000 samples using Latin hypercube sampling. From these samples, we tested most parameters of the system against the disease burden outcomes to determine which of those parameters were most influential. Note that some of these parameters are basic parameters, from which other parameters are derived. (For example, the number of individuals with AIDS is the total population size, times the HIV-prevalence, times 1–*h*_*h*_.

The results of this analysis can be compared to the analogous analysis from existing work focusing on a simpler model [20]: “Most of these correlations coincide with *a priori* expectations based on the role of each parameter in the model. The two parameters of which we take particular note are $\gamma_{+-}^{--}$ and *θ*_*/−*_. The sensitivity to *θ*_*/−*_(but not *θ* –_*/*_) indicates that intervention strategies with partially-adherent individuals may not be effective (and that marginal increases in the rate they drop off into untreated also has no effect), but that strategies targeting untreated individuals may be. The particular impact of $\gamma_{++}^{--}$ seems to be an artifact of the differential equations model, which assumes that if such individuals are treated with a directly-observed treatment, short course (DOTS) for TB successfully and recover more quickly, they contribute to a greater disease burden–presumably because in the HIV-negative group (the majority of the population) recovers quickly and may become ill again, with no preference to be treated again, potentially ending up in a longer-lasting untreated or partially-adherent state.”

The new sensitivity analysis reflects a greater accounting for longer-term behavior and the differentiation of bacteria types when compared with [20], with increased sensitivity to several parameters (*d*_*tb*_, $\gamma_{++}^{--},\;\gamma_{+-}^{--}$) in the low TB scenario without AIDS in Indonesia (a scenario of less interest), and increased sensitivity to *i*_*tb*_, *h*_*h*_, and *θ*_*– /*_ in the high TB scenario (with or without AIDS) in Indonesia (a significant change).

See Table 3 for all significant parameters. Beyond this summary, see S2 Appendix for the full sensitivity analysis, including a table of all parameters and variables.

**Table 3:** Sensitivity analysis summary. Table summarizing parameters to which disease burden is sensitive. Capital O denotes positive correlation (higher disease burden), while capital X denotes negative correlation. We have noted correlations above 0.1 (in absolute value), except in cases in Indonesia with high TB, where the threshold we use is 0.01. (This is because in a high TB scenario with a very large population, the spread of TB is almost entirely unrestricted–subject to far less influence of the dynamics of the immunocompromised sub-population, resulting in much smaller correlations and the need for a lesser threshold).

	*Swaziland*								*Indonesia*							
	Low TB				High TB				Low TB				High TB			
				No AIDS				No AIDS				No Aids				No AIDS
	I−^–^	I ^+ –^	I ^+ +^	I−^–^	I−^–^	I ^+ –^	I ^+ +^	I−^–^	I−^–^	I ^+ –^	I ^+ +^	I−^–^	I−^–^	I ^+ –^	I ^+ +^	I−^–^
*c_1_*												X				
*h_a_*	X		O		X		O	O		O	O		X			
*h_h_*		X	O			X	O		X	X				X		
*h_tb_*			O	O		O	O	O		O	O					
*i_tb_*	O				O						X		O	O	O	O
*d_tb_*												X				
*ρ^– –^*												O				
*ρ^++^*	O				O				O		O					
$\gamma_{++}^{--}$	O	O	O	O	O	O	O	O				X	O	O	O	O
$\gamma_{+-}^{--}$	X		X	X	X		X	X			X	X	X	X	X	X
*θ –_/_*											X	O				
*θ _/ –_*	X		X	X	X		X	X				X				
*β_0_*	O	O	O	O	O	O	O	O					O	O	O	O

## Discussion

### Swaziland

Chemoprophylaxis has long been considered to be a valuable infection management strategy among the HIV/AIDS-immunocompromised [64]. Despite the selective pressure favoring the emergence of resistance that is associated with its use [48], chemoprophylaxis is presumed to have had a net beneficial effect on opportunistic infection related morbidity and mortality. However, our model has demonstrated that this may not always be the case–especially within the developing world, where resource limitations and sociocultural factors such as nonbelief in the usefulness of antibiotics further complicate the health behavioral component of antimicrobial resistance [65–68]. For these reasons, both individual and population-level health remain crucial considerations to be addressed during the development of medical policy recommendations.

Given that 27.4% of Swaziland’s adult population is HIV/AIDS+, (making Swaziland the current world leader in HIV/AIDS prevalence) [22], we were hopeful that chemoprophylaxis could be counted upon to manage TB within that region. However, irrespective of the percentage of HIV/AIDS+ susceptibles receiving chemoprophylaxis, we observed a negligible factor reduction in TB-associated infectivity and mortality, as compared to the baseline condition of zero chemoprophylaxis use. These findings suggest that the use of chemoprophylaxis under conditions similar to those in Swaziland may not be benefitting HIV/AIDS patients to the extent expected. Moreover, we found that the effects of chemoprophylaxis can be overwhelmed by the presence of current infectives within the population. Our model indicates that even when 100% of HIV/AIDS+ susceptibles receive chemoprophylaxis, we observe only a negligible reduction in population-wide infectivity. The proportion of current infectives in the population, as well as those entering the infective category immediately via vertical transmission via vertical transmission (thereby bypassing the susceptible phase, during which they would receive chemoprophylaxis), reduce the benefits of chemoprophylaxis use.

The results of our initial work on the relationship between HIV/AIDS-prevalent host populations and antibiotic resistance demonstrated the potential for heightened emergence [20]. Due to this finding, we expected that the current model would reveal that chemoprophylaxis could further compound the risk of emergence–a catastrophic result, considering the protective benefits attributable to antibiotic chemoprophylaxis. Fortunately, despite the results of our previous work, we found that increased used of chemoprophylaxis by HIV/AIDS patients did not strongly select for drug resistance. Therefore, while chemoprophylaxis may fail to manage infection as effectively as expected in Swaziland, it is still provides some benefit to HIV/AIDS patients, without risking population-level public health by selecting for emergence of resistance.

### Indonesia

Among HIV/AIDS+ patients in Indonesia, the benefits of chemoprophylaxis were much more noticeable, with category-specific TB incidence declining by as much as 80% when 100% of HIV/AIDS+ susceptibles are treated. Given this decrease in observed incidence, we also noted a corresponding decrease in emergence of resistance and TB-associated mortality. These findings reflect the characteristics of the population: While pathogen persistence is typically constrained by the availability of susceptibles (as seen in Swaziland) [69, 70], Indonesia’s population of greater than 250 million, of whom less than 1% are TB infected [70], represents an effectively limitless reserve of susceptibles. Considering this condition, a high rate of contact and transmission is expected [71], especially in the absence of any mitigating host conditions. A decline in transmission, as would be expected due to the implementation of chemoprophylaxis regimens for HIV/AIDS patients, would, therefore, be impactful.

Despite these positive findings for HIV/AIDS patients, we must acknowledge that, contrary to Swaziland, the majority of TB cases in Indonesia occur among otherwise immunocompetent hosts[70]. This is a necessary consideration when assessing TB-associated risk in Indonesia. Again, while chemoprophylaxis does provide observable benefits to those affected by HIV/AIDS, it may not be an effective means of infection control for the Indonesian population as a whole. Rather, the combination of chemoprophylaxis availability to the majority (ideally 100%) of HIV/AIDS+ susceptibles, coupled with an early detection and treatment program such as DOTS [72] for those with active TB infections, may have the greatest impact on TB reduction under conditions similar to those in Indonesia.

## Conclusions

Having examined the risk of prophylaxis-associated emergence, versus the protective benefits it provides, we determined that the magnitude of its impact as an infection control measure varies depending on the prevalence of HIV/AIDS+ susceptibles within the population. In Swaziland, where more than a quarter of the population is HIV/AIDS+, and where 77% of TB cases occur in HIV/AIDS+ hosts [22, 23], the prevalence of current infectives compromises the intended effects of chemoprophylaxis. Even though TB transmission may be slightly curtailed as percent chemoprophylaxis treatment is increased, those who are already actively infective perpetuate transmission to the extent that only a negligible decrease in infectivity is observed–even when 100% of HIV/AIDS+ susceptibles are given chemoprophylaxis. Conversely, in Indonesia, where a significantly smaller percentage (0.46%) of the population is HIV/AIDS+ [21], and the majority of TB cases occur among the fully immunocompetent, the prophylaxis-associated benefits to HIV/AIDS+ susceptibles are observably greater.

These findings suggest that policy decisions regarding TB infection management should take into account the prevalence of both HIV/AIDS and HIV-incident TB in the target population, since the effectiveness of chemoprophylaxis is affected by both of these conditions. We also emphasize that, while chemoprophylaxis initially appears not to be contributing significantly to the rate of emergence of novel resistance, our follow up work will evaluate the impact of strain competition using a longer-term model.

## Supporting information

S1 Appendix Model description, equations, and parameters. Details of the model, including the abstract construction of the model, the equations derived from the abstraction, and the details (including value(s) used) of each parameter.
(DOCX)

S2 Appendix Sensitivity analysis. Complete details of the analysis of the disease burden (infective counts of each type) and its sensitivity to each parameter.
(DOCX)
