# Supplementary material for: Impact of chemorophylaxis policy for AIDS-immunocompromised patients on emergence of bacterial resistance
Source: PLoS One. 2020 Jan 30;15(1):e0225861. doi: 10.1371/journal.pone.0225861 (PMC6992000; doi:10.1371/journal.pone.0225861)
Supplement: S1 Appendix — Details of the model, including the abstract construction of the model, the equations derived from the abstraction, and the details (including value(s) used) of each parameter. (DOCX) [file pone.0225861.s001.docx]

**S1 Appendix. Model Description, Equations, and Parameters**

We present the description and details of this model in two parts. Because it is an extension of an the model in [1], we present that model first (as in the supplementary material to that manuscript) followed by the extension of the model to this more complex system that we have analyzed.

*Model Description: Simplified Model*

Our model examines the relative rate of emergence of antibiotic resistance in populations whose collective immunosuppression and prescribed antibiotic use patterns disrupt the selective pressures typically exerted on bacterial pathogens by host immune function and medically recommended antibiotic-taking behavior. We have chosen Indonesia and Swaziland as sample populations, since these countries represent the lower and upper extremes of HIV/AIDS prevalence within the developing world. The vast difference in HIV/AIDS prevalence that exists between the two countries (0.46% of the adult population in Indonesia is HIV/AIDS+, versus 27.4% in Swaziland[2]) suggests that there is a significant difference in the proportion of each population that is actively recommended to be taking antibiotics to treat or prevent infection.

Based on immune function alone, we recognize three categories of susceptible host: (1) those who are fully immunocompetent; (2) those rendered immunocompromised by active AIDS, including those incompletely adhering to highly active antiretroviral therapy (HAART); and, (3) those who are HIV/AIDS-positive, but whose consistent use of HAART provides them with a level of immune function sufficient to greatly reduce their risk of complications from AIDS-defining illness.[3, 4] Given the short-term nature of bacterial evolution (independent of the duration of infection), we chose to examine the emergence of antibiotic resistance over the course of 180 days. We therefore assume that no change in population description from seroconversion occurs;[3] immune status remains constant over time (though, future work will relax this assumption). Due to its high incidence in HIV/AIDS+ patients,[5] we parameterized our model with values reflecting tuberculosis, though the model can represent any bacterial infection.

We defined our susceptible, infectious and recovered populations according to four descriptors: immune status, HAART adherence, TB status, and antibiotic adherence. We denote immune (HIV/AIDS) and HAART using ^superscripts^, and TB status and adherence to antibiotics using _subscripts_ (Table A). For example, the compartment $S_{--}^{+-}$ is comprised of AIDS-immunocompromised, HAART-nonadherent hosts (^+-^ in the superscript), who are susceptible to bacterial infection, but are thus far infection-negative, and therefore do not take antibiotics (_--_ in the subscript).

|  | **Symbols** | **Definitions** |
| --- | --- | --- |
|  | -- | HIV/AIDS-, HAART- |
| **Superscripts** | +- | HIV/AIDS+, HAART- |
|  | ++ | HIV/AIDS+, HAART+ |
|  | -- | Infection-, untreated |
| **Subscripts** | ++ | Infection+, fully treated |
|  | +/ | Infection+, partially treated |
|  | +- | Infection+ , untreated |
|  | -+ | Infection-, prophylaxis |

**Table A. Key to Super- and Subscripts Used in the Model**

Within the ODE model, we describe variables using a combination of super- and subscripts reflecting immune and antibiotic treatment status. All possible combinations are shown.

In consideration of high HIV/TB co-infection prevalence – especially in the developing world[5-7] – we have chosen to use tuberculosis data to inform our parameter values, having derived those values directly from the literature whenever possible. We therefore use the work of Blower and Chou,[8] who estimate that fewer than 20% of TB cases are treated worldwide, and Trostle,[9] who reports a failure rate of 40-60% with respect to antibiotic adherence, to establish a relationship between C values when adherence is varied. When C_1_ is equal to 20%, we assume that C_2_ is equal to 50%, the mean of Trostle’s reported treatment failure rate. Under these circumstances, C_3_ is equal to 30%. In varying adherence, we maintain this initial ratio of probabilities by assuming that C_2_ is equal to 0.625*(1 – C_1_), and that C_3_ is equal to 0.375*(1 – C_1_).

We describe this scenario using the system of ordinary differential equations shown below, where the symbol $Î$ is used to represent the sum of all infectives that can that infect susceptibles at a rate of $\beta$, where $\beta$ depends on HIV/AIDS status of the susceptible, ζ represents the transition rate from exposed to actively infective, $\omega$ represents the HIV/AIDS excluded rate of death, $\omega_{A}$ represents the AIDS-attributable rate of death, $\omega_{I}$ represents the bacterial infection-attributable rate of death (as informed by tuberculosis), $\alpha$ represents per capita birthrate, $\rho$ represents the HIV/AIDS status-dependent rate of loss of immunity, $\gamma$ represents the HIV/AIDS and antibiotic category dependent rate of recovery from bacterial infection, $\theta$ represents the transition among antibiotic adherence states, and $\psi$ represents the HAART-dependent increase in infection-attributable death for patients with active AIDS. We have made the simplifying assumptions that HIV/AIDS+, HAART+ individuals give birth to HIV/AIDS- offspring, and that the offspring of HIV/AIDS+, HAART- individuals are also HIV/AIDS+. We expect the effects of these assumptions to be minor, especially given the few births likely to take place during the 180-day duration of the model. For detailed list of parameters, their condition dependencies, values used, and the reference from which they were estimated, see Table B.

| **Parameter Symbol** | **Description** | **Population** | **Value/Source** |
| --- | --- | --- | --- |
| N_IN_ | Total population | Indonesia | 253,609,643^[10]^ |
| N_SZ_ | Total population | Swaziland | 1,419,623^[2]^ |
| HA | HIV/AIDS prevalence | Indonesia | 0.46%^[10]^ |
|  |  | Swaziland | 27.4%^[2]^ |
| α | Daily birth rate | Indonesia | 4.67x10^-05^ ^[10]^ |
|  |  | Swaziland | 6.90x10^-05^ ^[2]^ |
| LE_A_ | Current population life expectancy at birth | Swaziland | 32 y^[2]^ |
| LE_HE_ | HIV-excluded life expectancy at birth | Swaziland | 61.5y^[2, 11]^ |
| ω | Daily HIV-excluded death rate | Indonesia | 3.69x10^-05 1^ |
|  |  | Swaziland | 4.45x10^-05 [2, 11]^ |
| ω_A_ | Daily death rate, attributable AIDS | Indonesia | 4.32x10^-05^ |
|  |  | Swaziland | 2.0x10^-03 2^ |
| ω_I_ | Daily tuberculosis-attributable death rate | Both | 8.22x10^-04 [12]^ |
| $\beta^{--}$ | Between-host bacterial transmission rate for fully immunocompetent hosts | Indonesia | 2.21x10^-05 [12, 13]^ |
|  |  | Swaziland | 7.9x10^-09 [12, 13]^ |
| $\beta^{+-}$ | Between-host bacterial transmission rate for HIV/AIDS+, HAART- | Indonesia | 1.72x10^-09^; assumed. |
|  |  | Swaziland | 4.64x10^-10^; assumed. |
| $\beta^{++}$ | Between-host bacterial transmission rate for HIV/AIDS+, HAART+ hosts | Indonesia | 9.49x10^-08 [12, 13]^ |
|  |  | Swaziland | 2.76x10^-09 [12, 13]^ |
| $\zeta^{--}$ | Rate of transition from exposed to infective, fully immunocompetent | Both | 3.10x10^-07[12]^ |
| $\zeta^{+-}$ | Rate of transition from exposed to infective, HIV/AIDS+, HAART- | Both | 3.41x10^-07^; assumed. |
| $\zeta^{++}$ | Rate of transition from exposed to infective, HIV/AIDS+, HAART+ | Both | 3.10x10^-07^; assumed. |
| $\rho^{--}$ | Rate of immune memory loss in fully immunocompetent hosts | Both | 1·0x10^-03^; assumed. |
| $\rho^{+-}$ | Rate of immune memory loss in HIV/AIDS+, HAART- hosts | Both | 1·0x10^-02^; assumed. |
| $\rho^{++}$ | Rate of immune memory loss in HIV/AIDS+, HAART+ hosts | Both | 2.0x10^-03^; assumed. |
| $\theta_{-/}$ | Transition rate from untreated to partially antibiotic adherent | Both | 1.05x10^-02 [14]^ |
| $\theta_{/-}$ | Transition rate from partially antibiotic adherent to untreated | Both | 1.05x10^-02[14]^ |
| $\gamma_{++}^{--}$ | TB recovery rate in HIV/AIDS-, DOTS-treated hosts | Both | 3.0x10^-03 [13, 15, 16]^ |
| $\gamma_{+/}^{--}$ | TB recovery rate in HIV/AIDS-, non-DOTS-treated hosts | Both | 2.0x10^-03 [13, 15, 16]^ |
| $\gamma_{+-}^{--}$ | TB recovery rate in HIV/AIDS-, untreated hosts | Both | 9.13x10^-04 [13, 15, 16]^ |
| $\gamma_{++}^{+-}$ | TB recovery rate in HIV/AIDS+, HAART-, DOTS-treated hosts | Both | 2.0x10^-03 [13, 15, 16]^ |
| $\gamma_{+/}^{+-}$ | TB recovery rate in HIV/AIDS+, HAART-, non-DOTS-treated hosts | Both | 1.5x10^-03 [13, 15, 16]^ |
| $\gamma_{+-}^{+-}$ | TB recovery rate in HIV/AIDS+, HAART-, untreated hosts | Both | 3.0x10^-04 [13, 15, 16]^ |
| $\gamma_{++}^{++}$ | TB recovery rate in HIV/AIDS+, HAART+, DOTS-treated hosts | Both | 1.50x10^-4^; assumed. |
| $\gamma_{+/}^{++}$ | TB recovery rate in HIV/AIDS+, HAART+, non-DOTS-treated hosts | Both | 1.0x10^-3^; assumed. |
| $\gamma_{+-}^{++}$ | TB recovery rate in HIV/AIDS+, HAART+, untreated hosts | Both | 4.57x10^-4^; assumed. |
| C_1_ | Probability of early TB detection and complete (DOTS) treatment | Both | Varied, depending on percent complete adherence^[8]^ |
| C_2_ | Probability of incomplete (non-DOTS) TB treatment | Both | 0.625* (1-C_1_)^[9]^ |
| C_3_ | Probability that no treatment was ever sought | Both | 0.375* (1-C_1_); assumed. |
| $\psi^{+-}$ | Multiplier representing increased risk of infection death in HIV/AIDS+, HAART- infectives | Both | 3.3^[17]^ |
| $\psi^{++}$ | Factor of increase for infection-attributable death in HIV/AIDS+, HAART+ patients | Both | 1 (placeholder only). |

**Table B. SEIR Model Parameter Values**

Table B defines the values for each parameter used in the model; some parameter values were readily available in the literature, while others were calculated using known values, with the formulas corresponding to these calculations appearing in the supplement. Where applicable, we assume equivalent immune function in HIV/AIDS- and HIV/AIDS+, HAART+ hosts. We note that, rather than the annual figures typically reported in the literature, parameter values for this model have been converted into per person, per day probabilities to reflect the duration of the model. HIV-excluded and AIDS-attributable mortality in Indonesia were adjusted using current and past mortality rates for Swaziland. The models used for this process appear in the appendix to this paper. The model used to separate AIDS-attributable mortality from all-cause mortality is located in the supplement.

$$\frac{dS_{--}^{--}}{dt}= -\beta^{--}S_{--}^{--}Î-\omega S_{--}^{--}+\alpha(S_{--}^{--}+ S_{--}^{++}+ E_{--}^{--}+ E_{--}^{++}+I_{++}^{--}+I_{++}^{++}+R_{--}^{--}+R_{--}^{++})+\rho^{--}R_{--}^{--}$$

$$\frac{dE_{--}^{--}}{dt}=\beta^{--}S_{--}^{--}Î-\omega E_{--}^{--}- \zeta^{--}E_{--}^{--}$$

$$\frac{d I_{++}^{--}}{dt}=\zeta^{--}E_{--}^{--}C_{1}- \gamma_{++}^{--}I_{++}^{--}-\omega I_{++}^{--}$$

$$\frac{dI_{+/}^{--}}{dt}=\zeta^{--}E_{--}^{--}C_{2}- \gamma_{+/}^{--}I_{+/}^{--}- I_{+/}^{--} \left( \omega+ \omega_{I} \right)+ \alpha I_{+/}^{--}+\theta_{+/}I_{+-}^{--}- \theta_{+-}I_{+/}^{--}$$

$$\frac{dI_{+-}^{--}}{dt}=\zeta^{--}E_{--}^{--}C_{3}- \gamma_{+-}^{--}I_{+-}^{--}- I_{+-}^{--}\left( \omega+ \omega_{I} \right)+ \alpha I_{+-}^{--}-\theta_{+/}I_{+-}^{--}+ \theta_{+-}I_{+/}^{--}$$

$$\frac{dR_{--}^{--}}{dt}= \gamma_{++}^{--}I_{++}^{--}+ \gamma_{+/}^{--}I_{+/}^{--}+\gamma_{+-}^{--}I_{+-}^{--} - \rho^{--}R_{--}^{--}$$

$$\frac{dS_{--}^{+-}}{dt}= -\beta^{+-}S_{--}^{+-}Î- S_{--}^{+-}\left( \omega+\omega_{A} \right)+ \alpha\left( S_{--}^{+-}+E_{--}^{+-}+ I_{++}^{+-}+ R_{--}^{+-} \right)+ \rho^{+-}R_{--}^{+-}$$

$$\frac{dE_{--}^{+-}}{dt}= \beta^{+-}S_{--}^{+-}Î- E_{--}^{+-}\left( \omega_{A}+ \omega_{I} \right)-\zeta^{+-}E_{--}^{+-}$$

$$\frac{d I_{++}^{+-}}{dt}= \zeta^{+-}E_{--}^{+-}C_{1}- \gamma_{++}^{+-}I_{++}^{+-}-I_{++}^{+-}(\omega+\psi^{+-}\omega_{I}+\omega_{A})$$

$$\frac{dI_{+/}^{+-}}{dt}= \zeta^{+-}E_{--}^{+-}C_{2}- \gamma_{+/}^{+-}I_{+/}^{+-}-I_{+/}^{+-}\left( \omega+\psi^{+-}\omega_{I}+\omega_{A} \right)+\alpha I_{+/}^{+-}- \theta_{+-}I_{+/}^{+-}+ \theta_{+/}I_{+-}^{+-}$$

$$\frac{dI_{+-}^{+-}}{dt}= \zeta^{+-}E_{--}^{+-}C_{3}- \gamma_{+-}^{+-}I_{+-}^{+-}-I_{+-}^{+-}\left( \omega+\psi^{+-}\omega_{I}+\omega_{A} \right)+\alpha I_{+-}^{+-}-\theta_{+/}I_{+-}^{+-}+ \theta_{+-}I_{+/}^{+-}$$

$$\frac{d R_{--}^{+-}}{dt}= \gamma_{++}^{+-}I_{++}^{+-}+ \gamma_{+/}^{+-}I_{+/}^{+-}+ \gamma_{+-}^{+-}I_{+-}^{+-}- R_{--}^{+-}\left( \omega+ \omega_{A} \right)- \rho^{+-}R_{--}^{+-}$$

$$\frac{d S_{--}^{++}}{dt}= -\beta^{++}S_{--}^{++}Î- {\omega S}_{--}^{++}+ \rho^{++}R_{--}^{++}$$

$$\frac{d E_{--}^{++}}{dt}= -\beta^{++}S_{--}^{++}Î- {\omega E}_{--}^{++}-\zeta^{++}E_{--}^{++}$$

$$\frac{dI_{++}^{++}}{dt}=\zeta^{++}E_{--}^{++}C_{1}- \gamma_{++}^{++}I_{++}^{++}-\omega I_{++}^{++}$$

$$\frac{dI_{+/}^{++}}{dt}= \zeta^{++}E_{--}^{++}C_{2}- \gamma_{+/}^{++}I_{+/}^{++}-I_{+/}^{++}\left( \omega+\psi^{++}\omega_{I} \right)+\alpha I_{+/}^{++}- \theta_{+-}I_{+/}^{++}+ \theta_{+/}I_{+-}^{++}$$

$$\frac{dI_{+-}^{++}}{dt}= \zeta^{++}E_{--}^{++}C_{3}- \gamma_{+-}^{++}I_{+-}^{++}-I_{+-}^{++}\left( \omega+{\psi^{++}\omega}_{I} \right)+ \alpha I_{+-}^{++}+\theta_{+-}I_{+/}^{++}- \theta_{+/}I_{+-}^{++}$$

$$\frac{dR_{--}^{++}}{dt}= \gamma_{++}^{++}I_{++}^{++}+ \gamma_{+/}^{++}I_{+/}^{++}+ \gamma_{+-}^{++}I_{+-}^{++}- {\omega R}_{--}^{++}- \rho^{++}R_{--}^{++}$$

*Calculation of Example Parameter Values*

To reflect the entry and re-entry of new susceptibles into the population via birth and gradual loss of immune memory, respectively, as well as the departure, due to death, of individuals from all compartments (even just within the relatively short 180 day duration of the model), we defined several parameters (Table B). (Where possible, the parameter values used in this model were taken from the existing literature; when we were unable to do this, values were assumed, or calculated using the models detailed in this section.) Mortality-associated parameter values include separate rates for HIV-excluded, bacterial infection-related, and other-cause AIDS-related death. We also use the parameters $\rho$ and $\theta$, which represent the differing rate of immune memory loss for each HIV status, and the rate of transition between antibiotic adherence categories, respectively. We note that ρ, β, and ζ (the transition rate from exposed to infective) depend on immune status only, and do not vary based on antibiotic adherence; the opposite is true for the parameter θ, which varies based on antibiotic adherence alone. Parameters such as these, whose values are assigned based on either HIV/AIDS status or antibiotic adherence, but not both, are singly indexed using the applicable sub- or superscript.

Although we use $\rho$ to represent the rate at which immune memory is lost during recovery from a bacterial infection, we recognize that it is difficult, if not impossible, to assign a value to a parameter that essentially equates to the number of T-cells lost per day.[18] Because we cannot ignore the difference in immune memory that exists between immunocompetent and AIDS-immunocompromised hosts,[19] we assigned an arbitrary value of 0.001 to represent rate of immune memory loss among the immunocompetent. We assume that immune memory is lost twice as fast in HIV/AIDS+, HAART-treated hosts[20] and ten times as fast in those with active AIDS. (In assigning parameter values to HIV/AIDS+, HAART-adherent hosts, we assume their immunocompetence to be equivalent to that HIV/AIDS- hosts in all instances except with regard to loss of immune memory.) Though these particular values are chosen arbitrarily, the qualitative outcome of the model will be unaffected so long as the values remain monotonically increasing.

Due to financial constraints, people in developing countries may forgo antibiotic treatment[21-23] (possibly in favor of purchasing HAART), but, we found no data indicating the frequency with which they alternate between partially adherent and untreated. However, using the work of Kaona, et al.,[14] we were able to establish a mean duration of adherence to antibiotic treatment in TB+ hosts by taking a weighted mean of the points at which patients ceased treatment prior to finishing their antibiotic regimens. We use the inverse of that 95.1-day duration to represent the rate at which infectives transition between the partially adherent and untreated states.

We use the symbol ζ to represent the rate of transition from exposure to active infectivity. Cohen and Murray[12] provide an annual transition rate, which we convert to reflect a daily value, and use as a baseline for fully immunocompetent and HAART+ hosts. We assume that those with active AIDS transition ten-percent more quickly once exposed.

In keeping with the primary literature on compartmental modeling, we use the parameter β to represent the between-host bacterial transmission rate; and we use γ to represent the rate of recovery once a bacterial infection is contracted.[24, 25] We assume that β is equivalent for HIV- and HIV/AIDS+, HAART-adherent individuals, whereas the β value for AIDS+, HAART-nonadherent individuals was assumed to be ten-percent higher than that of their fully immunocompetent and HAART-adherent comparators. In all cases, we rely on the work of Cohen and Murray,[12] who assigned an arbitrary annual transmission rate of 8.50x10^-6^ to their study population of one million. However, we converted this annual transmission rate to a daily transmission rate, and adjusted it to account for differing population sizes in Indonesia and Swaziland, and differing subpopulation sizes among immune classes.

Values for γ were assigned based on previously published TB infection duration data. Dye, et al.,[26] report that in fully treated, immunocompetent hosts, TB infection lasts approximately 292 days; whereas, with partial treatment, approximate infection duration is 547.5 days. Tiemersma, et al.,[16] estimate an infection duration of 1095 days in the complete absence of antibiotic treatment. We use the inverse of these three durations to establish baseline rates of recovery in fully immunocompetent hosts.

For HIV/AIDS+, HAART+ hosts, we assume that each antibiotic-dependent value of γ is equivalent to 50% of the one corresponding to fully immunocompetent hosts of the same antibiotic status. We made this assumption to reflect the potential immune complications that can arise within this subpopulation, despite HAART treatment, and to reflect a more reasonable steady state for the ratio of β to γ. In all cases, these values are reflective of initial values used in Cohen[12] and Dye.[13, 15]

For AIDS-immunocompromised hosts, we the use the tuberculosis case fatality rates (CFRs) set forth by Corbett, et al.,[17] to modify estimated rates of recovery in hosts lacking immune function. Corbett reports a mean CFR ratio of 1.5 for HIV/AIDS+ versus immunocompetent hosts with full (DOTS) antibiotic treatment.[17, 27] (Corbett’s work separates CFRs according to smear-negative and smear-positive samples. However, since our work does not address smear- vs. smear+ TB, we apply a mean of the smear- and smear+ CFRs to our baseline γ values to adjust for immunoincompetence due to active AIDS.) We apply the inverse of that ratio, two-thirds, to $\gamma_{++}^{--}$ – with the assumption that being 1.5 times more likely to succumb to tuberculosis is the equivalent of being 0.67 times as likely to recover – in order to define a recovery rate for fully antibiotic adherent, HIV/AIDS+, HAART- hosts. Similarly, we apply the inverse of 1.27[17] to $\gamma_{+/}^{--}$ to adjust for immunoincompetence in partially treated (non-DOTS), HIV/AIDS+, HAART- hosts; and, in the complete absence of antibiotic treatment, we apply the inverse of 3.3[17] to $\gamma_{+-}^{--}$, to calculate a rate of recovery in AIDS-immunocompromised hosts.

We apply the multiplier $\psi$to the parameter $\omega_{I}$ to account for the increased probability of infection-attributable mortality affecting HIV/AIDS+, non-HAART-adherent hosts. We again refer to Corbett, et al.,[17] and assume a worst-case scenario, in which infection-attributable mortality is 3.3 times greater than it is in fully immunocompetent and HAART-treated hosts. Accordingly, we set $\psi^{+-}$ equal to 3.3 for those AIDS+ infectives not receiving HAART. We assume that immunocompetence and/or HAART is largely protective against death from opportunistic infection in antibiotic-adherent infectives, so we assign a value of one to $\psi^{++}$. (Values for ψ are based on HIV status alone.)

*Antibiotic Adherence*

The literature reports ranges of antibiotic adherence from 5-95%, depending on factors such as age, socioeconomic status and medication side-effects.[28-30] We investigated the impact of adherence on emergence using a range of 0-100% adherence. When comparing projected emergence both with and without HIV/AIDS in the population, we also included a trial in which we assumed all categories of hosts were 20% adherent to reflect the best-case scenario of adherence reported by Blower and Chou.[8]

*Per Host Category Relative Emergence Calculations*

Determining the relative emergence attributable to each host category required that we include estimates of the per cell, per bacterial generation mutation rate; the total number of infected cells per host; the expected number of bacterial generations per infection duration; the per category infection duration; and the relative success of the mutant strain (Table C).

| **Infective Category** | **Per cell, Per Generation, Mutation Rate** | **Total Infected Cells per Host** | **Per Category Infection Duration (Days)** | **Generations Per Infection Duration** | **Relative Success of Mutant** |
| --- | --- | --- | --- | --- | --- |
| $I_{++}^{--}$ | 3.00x10^-10[31]^ | 2.08x10^5^ | 292 | 194.67 | 0.95 |
| $I_{+/}^{--}$ | 2.25x10^-10^ | 3.11x10^5^ | 547 | 365 | 0.95 |
| $I_{+-}^{--}$ | 1.50x10^-10^ | 4.15x10^5[32, 33]^ | 1095 | 730 | 0.05 |
| $I_{++}^{+-}$ | 3.30x10^-10^ | 2.59x10^5^ | 438 | 292 | 0.95 |
| $I_{+/}^{+-}$ | 2.48x10^-10^ | 3.89x10^5^ | 695 | 463 | 0.95 |
| $I_{+-}^{+-}$ | 1.65x10^-10^ | 5.19x10^5^ | 3613 | 2409 | 0.05 |
| $I_{++}^{++}$ | 3.00x10^-10[31]^ | 2.08x10^5^ | 292 | 194 | 0.95 |
| $I_{+/}^{++}$ | 2.25x10^-10^ | 3.11x10^5^ | 547 | 365 | 0.95 |
| $I_{+-}^{++}$ | 1.50x10^-10^ | 4.15x10^5[32, 33]^ | 1095 | 730 | 0.05 |

**Table C. Projected Emergence Parameters**

To calculate relative projected emergence attributable to each host category, we estimated relevant bacteriological rates. Generations per infection duration were estimated based on the work of Gill,[34] and previously discussed γ values;[16, 17, 26] whereas the relative success of mutations was arbitrarily assigned based on selective pressures exerted by host health behavior and immune function.[35-37]

*Per cell, Per Bacterial Generation Mutation Rate*

To estimate this parameter value, we relied upon the work Billington, et al.,[31] who report a range of 2.0x10^-10^ to 4.0x10^-10^ mutations per cell, per generation, in *Mycobacterium tuberculosis* (MTB) in the presence of Rifampin. Using the mean of that range, we establish a baseline mutation rate of 3.0x10^-10^ for fully immunocompetent (and HAART+), antibiotic-adherent hosts. In the absence of the selective pressure applied by antibiotic use,[38] we assume mutations to develop 50% less frequently in immunocompetent and HAART+, antibiotic-negative hosts, as compared to the baseline. Finally, among partially-treated hosts, we expect that some selective pressure will be exerted by the intermittent use of antibiotics; however, we do not expect that mutations will arise as frequently as they do in fully-adherent hosts. (Rather, we expect that drug-resistant strains already present within the host microbiome will gain a competitive advantage under this condition.) We therefore use the mean of the mutation rates for fully-adherent and untreated hosts to establish a mutation rate of 2.25x10^-10^ for partially-treated HIV/AIDS- and HIV/AIDS+, HAART+, hosts.

Among AIDS-immunocompromised hosts, the selective pressure typically applied by a functional immune response is absent.[39, 40] While this might suggest that fewer mutations would occur, bacterial replication occurs more rapidly in immunocompromised hosts,[41] thereby presumably increasing the probability of random mutation. Therefore, for each antibiotic adherence category, we assume an arbitrary 10% increase in the per bacterium mutation rate of AIDS-immunocompromised hosts, relative to their fully immunocompetent and HAART+ comparators.

*Total Infected Cells per Host*

To calculate an expected number of infected cells for each host category, we referenced Stone, et al.,[33] and Dormans, et al.,[32] who provide estimates for the total number of alveolar cells in the mouse and human lung, and the number of MTB colony-forming units (CFUs) found in a mouse model, respectively. In examining strain-specific MTB colonization within the lungs of a mouse host, Dormans, et al., found a range of approximately 10^5^-10^9^ CFUs.[32] Assuming that MTB resides solely in the cells of alveolar region, and assuming a best-case bacillary load of 10^5^ CFUs, we divided 10^5^ by the total number of alveolar cells present in the mouse[33] to establish a percentage of infected cells. We then multiplied that same percentage by the total number of alveoli present in human lungs[33] to correct for the large difference in total alveolar cells that exists between the mouse and human. This process produced a baseline of 4.15x10^5^ infected alveoli in fully immunocompetent and HAART-adherent hosts. We assumed that, among fully-treated hosts, 50% fewer infective alveoli would be found; and we used the mean number of infective alveoli found in fully-treated and untreated hosts to estimate infective alveoli in those who are partially-treated. Finally, among the AIDS-immunocompromised, we assumed a 25% increase in infected alveoli in fully and untreated hosts, relative to their immunocompetent counterparts. We then used the mean infective alveoli form those host categories to establish an estimate for partially treated hosts. (While we recognize that this process only provides us with a rough estimate of the number of infected alveoli, we do not expect it to significantly affect our results, nor do we expect it to compromise our ability to determine relative emergence attributable to each category of infective host.)

*Bacterial Generations per Infection Duration*

The expected number of bacterial generations per infection duration for each host category was estimated using Gill et al.,[34] who report that MTB doubling time ranges from 18-54h. Accordingly, we assume an average doubling time of 36h. For each adherence category, we apply that doubling time in the equation (1/γ*24h/1d)/36h, wherein 1/γ represents the per category infection duration.

*Relative Probability of Mutant Success*

Based on the combination of HIV/AIDS status and antibiotic adherence, each category of infective was assigned a value representing the relative probability that an antibiotic-resistant mutant would arise and successfully compete for host resources. These probability values were assumed based on the selective pressures associated with each host category. For example, emergence of antibiotic resistance is less likely in immunocompetent, antibiotic adherent hosts, but those mutations that do arise face little host resource competition,[37] since the combination of host immune function and appropriate antibiotic use exerts a twofold selective pressure against drug-sensitive strains.[36] The probability of evolutionary success of a drug-resistant mutant is, therefore, presumed to be high for the host category $I_{++}^{--}$. Accordingly, the probability value assigned to that host category is 0.95. Conversely, antibiotic-untreated, AIDS-immunocompromised, hosts lack the selective pressures typically applied by immune function and antibiotic adherence against drug-sensitive pathogens; drug-resistant strains are, therefore, unlikely to arise by means other than random mutation and horizontal transfer from antibiotic-resistant microbes already present within the host microbiome.[35]

*AIDS-attributable Mortality Adjustments*

In order to accurately estimate a death rate due to attributable AIDS, it was necessary to divide the reported HIV/AIDS prevalence for each population into two separate groups: those who are HIV+, but whose disease has not yet progressed to AIDS, and those who are AIDS+. Morgan, et al.,[11] report a median progression time from HIV seroconversion to active AIDS of 3431 days, and a median survival time from AIDS to death of 276 days in the rural Africa during the pre-HAART era; the total post-seroconversion survival time is 3707 days, 276 days of which are spent in the AIDS+ category. That is, of the total post-seroconversion survival time, 93% is spent with HIV only, and 7% is spent with active AIDS. Accordingly, we estimate that of the HIV/AIDS+ hosts present in each country, 93% are HIV+, and 7% have active AIDS.

Since our model reflects the current state of HAART availability, it was necessary to adjust AIDS-attributable death to account for the longer post-seroconversion lifespans made possible by the introduction of antiretrovirals.[42] However, an updated estimate of post-activation of AIDS survival time in HAART-treated hosts was not readily available in the literature. We therefore adjust for the additional total post-seroconversion survival time afforded by HAART availability, while assuming that the same percentages apply to time spent in each category as were estimated using Morgan;[11] we expect that this assumption may change the outcome of the model slightly, but not significantly.

The model below, the parameter symbols for which appear in Table D, was used to calculate the attributable AIDS death rate for Swaziland:

1/ ST_ASZ_ = (1/ LE_SZ_ – 1/ LE_HESZ_*IC_PSZ_)/A_PSZ_

Solving for ST_ASZ_, and taking its inverse provides an annual AIDS-attributable death rate, and we divide that number by 365 to determine ω_A_, the daily rate.

| **Parameter Symbol** | **Description** | **Value/Source** |
| --- | --- | --- |
| N_SZ_ | Total population, Swaziland | 1,419,623^[2]^ |
| HA_SZ_ | HIV/AIDS prevalence | 26.5%^[2]^ |
| LE_SZ_ | Current population life expectancy at birth | 32 y^[43]^ |
| LE_HESZ_ | HIV-excluded life expectancy at birth | 61.5 y^[42]^ |
| ω_ASZ_ | AIDS attributable death rate (d) | (1/ ST_ASZ_)/365 |
| P_HIV_ | Proportion of HIV/AIDS+ hosts with HIV only | 0.93^[11]^ |
| P_AIDS_ | Proportion of HIV/AIDS+ hosts with active AIDS | 0.07^[11]^ |
| A_POPSZ_ | People living with active AIDS, Swaziland | N_SZ_* HA_SZ_ * P_AIDS_ |
| IC_POPSZ_ | People who are fully immunocompetent or HAART treated, Swaziland | N_SZ_ - A_POPSZ_ |
| IC_PSZ_ | Percent of population that is fully immunocompetent or HAART treated, Swaziland | IC_POPSZ_/NSZ |
| A_PSZ_ | Percent of population that with active AIDS, Swaziland | A_POPSZ_/NSZ |

**Table D. AIDS-attributable mortality estimates, Swaziland**

The parameters detailed above were used to estimate an AIDS-attributable mortality rate which applies only to those Swazilanders with active AIDS. We assume that HIV/AIDS+, HAART-adherent, hosts are susceptible only to HIV-excluded and infection-related death.

In Indonesia, calculating the AIDS-attributable death rate required that we adjust for the fact that population life expectancy is now greater than it was prior to the introduction of HIV/AIDS. (This phenomenon, actually the result of medical advances unrelated to HIV/AIDS,[44] masks the impact of HIV/AIDS on life expectancy.) We use the model described below to adjust for increased Indonesian life expectancy in the post-HIV/AIDS era. Parameter values and symbols are found in Tables D and E.

| **Parameter Symbol** | **Description** | **Value/Source** |
| --- | --- | --- |
| N_SZ_ | Total population, Swaziland | 1,419,623^[2]^ |
| N_IN_ | Total population, Indonesia | 253,609,643^[10]^ |
| HA_SZ_ | HIV/AIDS prevalence, Swaziland | 26.5%^[2]^ |
| HA_IN_ | HIV/AIDS prevalence, Indonesia | 0.4%^[10]^ |
| LE_SZ_ | Current population life expectancy at birth, Swaziland | 32 y^[43]^ |
| LE_IN_ | Current population life expectancy at birth, Indonesia | 72.2 y^[10]^ |
| LE_SZ80_ | Pre-AIDS life expectancy, Swaziland | 61.5 y^[42]^ |
| LE_IN80_ | Pre-AIDS life expectancy, Indonesia | 52.5y ^[45]^ |
| LE_HESZ_ | Current HIV-excluded life expectancy, Swaziland | 61.5 y^[42]^ |
| LE_HEIN_ | Current HIV-excluded life expectancy, Indonesia | 74.3y |
| ω_A’_ | Adjusted AIDS attributable death rate (d), Swaziland | (1/ ST_ASZ’_)/365 |
| P_HIV_ | Proportion of HIV/AIDS+ hosts with HIV only | 0.93^[11]^ |
| P_AIDS_ | Proportion of HIV/AIDS+ hosts with active AIDS | 0.07^[11]^ |
| A_POPSZ_ | People living with active AIDS, Swaziland | N_SZ_* HA_SZ_ * P_AIDS_ |
| IC_POPSZ_ | People who are fully immunocompetent or HAART treated, Swaziland | N_SZ_ - A_POP_ |
| IC_PSZ_ | Percent of population that is fully immunocompetent or HAART treated, Swaziland | IC_POPSZ_/NSZ |
| A_PSZ_ | Percent of population with active AIDS, Swaziland | A_POPSZ_/NSZ |
| A_POPIN_ | People living with active AIDS, Indonesia | N_IN_*HA_IN_*P_AIDS_ |
| IC_POPIN_ | People who are fully immunocompetent or HAART treated, Indonesia | N_IN -_ A_POPIN_ |
| IC_PIN_ | Percent of population that is fully immunocompetent or HAART-treated, Indonesia | IC_POPIN_/N_IN_ |
| A_PIN_ | Percent of population with active AIDS, Indonesia | A_POPIN_/N_IN_ |

**Table E. AIDS-attributable mortality, India**

AIDS-attributable mortality for Indonesia was calculated based on an adjusted life expectancy for Swaziland. This allowed us to avoid the appearance that HIV/AIDS is adding life-years to population life expectancy when, in fact, medical advances unrelated to HIV/AIDS are the reason for longer lifespans.

Using the equation 1/ST_ASZ_ = (1/ LE_SZ_ - (1/LE_SZ80_* IC_PSZ_))/A_PSZ_, we solve for an adjusted survival time with active AIDS in Swaziland and apply its inverse to the ratio of pre-AIDS life expectancies in Swaziland and Indonesia to determine HIV-excluded life expectancy in Indonesia as follows:

1/LE_HEIN_ = (1/LE_IN_ - 1/ST_ASZ’_*(LE_SZ80_/LE_IN80_) *(A_PIN_))/ IC_PIN_

Finally, to calculate death due to AIDS in Indonesia, we apply 1/LE_HEIN_ to the ratio LE_SZ80_/LE_IN80_, and divide the product by 365 to assess daily AIDS-related mortality.

We note that this model relies upon two assumptions: First, we assume that the post-AIDS life expectancy ratio in Indonesia and Swaziland is equivalent to the pre-AIDS life expectancy ratio; and, second, we assume that life expectancy among the immunocompetent in Swaziland hasn’t changed between the current and pre-AIDS era.

*Model Description: Extended Model*

We duplicate the descriptive system used in Chapter 1 in this model. As such, we use a ^superscript^ to dually describe HIV and HAART status; and we use a _subscript_ to describe both bacterial infection status and adherence to antibiotics. We also introduce the subscript combination “+-” to describe infection-negative, prophylaxis-positive, susceptibles, thereby distinguishing them from hosts using antibiotics to treat active infection.

The entire system is described by a set of ordinary differential equations where the symbol $Î$ is used to represent the sum of all infectives that can that infect susceptibles at a rate of $\beta$, where $\beta$ depends on immune status, ζ, which represents the immune status-dependent rate of transition from exposed to actively infective, $\omega_{I}$ represents rate of death due to bacterial infection (as informed by TB data), $\omega_{A}$ represents AIDS-attributable rate of death, ω all other cause-related rate of death, $\alpha$ represents per capita birthrate, $\rho$ represents the immune status-dependent rate of loss of immunity, $\gamma$ represents the HIV/AIDS and antibiotic category dependent rate of recovery from bacterial infection, $\theta$ represents the rate of transition between the partially antibiotic adherent and untreated states; and $\psi$ represents the HAART-dependent increase in infection-attributable death for patients with active AIDS.

$$\frac{dS_{-+}^{+-}}{dt}= -\beta^{+-}S_{-+}^{+-}Î- S_{-+}^{+-}\left( \omega+\omega_{A} \right)+ \alpha S_{-+}^{+-}$$

$$\frac{dE_{-+}^{+-}}{dt}= \beta^{+-}S_{-+}^{+-}Î- E_{-+}^{+-}\left( \omega+\omega_{A} \right)- \zeta E_{-+}^{+-}$$

$$\frac{d S_{-+}^{++}}{dt}= -\beta^{++}S_{-+}^{++}Î- {\omega S}_{-+}^{++}+ \alpha S_{-+}^{++}$$

$$\frac{dE_{-+}^{++}}{dt}= \beta^{++}S_{-+}^{++}Î- E_{-+}^{++}\left( \omega+\omega_{A} \right)- \zeta E_{-+}^{++}$$

$$\frac{dS_{--}^{--}}{dt}= -\beta^{--}S_{--}^{--}Î-\omega S_{--}^{--}+\alpha(S_{--}^{--}+ S_{--}^{++}+ E_{--}^{--}+ E_{--}^{++}+I_{++}^{--}+I_{++}^{++}+R_{--}^{--}+R_{--}^{++})+\rho^{--}R_{--}^{--}$$

$$\frac{dE_{--}^{--}}{dt}=\beta^{--}S_{--}^{--}Î-\omega E_{--}^{--}- \zeta^{--}E_{--}^{--}$$

$$\frac{d I_{++}^{--}}{dt}=\zeta^{--}E_{--}^{--}C_{1}- \gamma_{++}^{--}I_{++}^{--}-\omega I_{++}^{--}$$

$$\frac{dI_{+/}^{--}}{dt}=\zeta^{--}E_{--}^{--}C_{2}- \gamma_{+/}^{--}I_{+/}^{--}- I_{+/}^{--} \left( \omega+ \omega_{I} \right)+ \alpha I_{+/}^{--}+\theta_{+/}I_{+-}^{--}- \theta_{+-}I_{+/}^{--}$$

$$\frac{dI_{+-}^{--}}{dt}=\zeta^{--}E_{--}^{--}C_{3}- \gamma_{+-}^{--}I_{+-}^{--}- I_{+-}^{--}\left( \omega+ \omega_{I} \right)+ \alpha I_{+-}^{--}-\theta_{+/}I_{+-}^{--}+ \theta_{+-}I_{+/}^{--}$$

$$\frac{dR_{--}^{--}}{dt}= \gamma_{++}^{--}I_{++}^{--}+ \gamma_{+/}^{--}I_{+/}^{--}+\gamma_{+-}^{--}I_{+-}^{--} - \rho^{--}R_{--}^{--}$$

$$\frac{dS_{--}^{+-}}{dt}= -\beta^{+-}S_{--}^{+-}Î- S_{--}^{+-}\left( \omega+\omega_{A} \right)+ \alpha\left( S_{--}^{+-}+E_{--}^{+-}+ I_{++}^{+-}+ R_{--}^{+-} \right)+ \rho^{+-}R_{--}^{+-}$$

$$\frac{dE_{--}^{+-}}{dt}= \beta^{+-}S_{--}^{+-}Î- E_{--}^{+-}\left( \omega_{A}+ \omega_{I} \right)-\zeta^{+-}E_{--}^{+-}$$

$$\frac{d I_{++}^{+-}}{dt}= \zeta^{+-}E_{--}^{+-}C_{1}+\zeta E_{-+}^{+-}- \gamma_{++}^{+-}I_{++}^{+-}-I_{++}^{+-}(\omega+\psi^{+-}\omega_{I}+\omega_{A})$$

$$\frac{dI_{+/}^{+-}}{dt}= \zeta^{+-}E_{--}^{+-}C_{2}- \gamma_{+/}^{+-}I_{+/}^{+-}-I_{+/}^{+-}\left( \omega+\psi^{+-}\omega_{I}+\omega_{A} \right)+\alpha I_{+/}^{+-}- \theta_{+-}I_{+/}^{+-}+ \theta_{+/}I_{+-}^{+-}$$

$$\frac{dI_{+-}^{+-}}{dt}= \zeta^{+-}E_{--}^{+-}C_{3}- \gamma_{+-}^{+-}I_{+-}^{+-}-I_{+-}^{+-}\left( \omega+\psi^{+-}\omega_{I}+\omega_{A} \right)+\alpha I_{+-}^{+-}-\theta_{+/}I_{+-}^{+-}+ \theta_{+-}I_{+/}^{+-}$$

$$\frac{d R_{--}^{+-}}{dt}= \gamma_{++}^{+-}I_{++}^{+-}+ \gamma_{+/}^{+-}I_{+/}^{+-}+ \gamma_{+-}^{+-}I_{+-}^{+-}- R_{--}^{+-}\left( \omega+ \omega_{A} \right)- \rho^{+-}R_{--}^{+-}$$

$$\frac{d S_{--}^{++}}{dt}= -\beta^{++}S_{--}^{++}Î- {\omega S}_{--}^{++}+ \rho^{++}R_{--}^{++}$$

$$\frac{d E_{--}^{++}}{dt}= -\beta^{++}S_{--}^{++}Î- {\omega E}_{--}^{++}-\zeta^{++}E_{--}^{++}$$

$$\frac{dI_{++}^{++}}{dt}=\zeta^{++}E_{--}^{++}C_{1}+\zeta E_{-+}^{++}- \gamma_{++}^{++}I_{++}^{++}-\omega I_{++}^{++}$$

$$\frac{dI_{+/}^{++}}{dt}= \zeta^{++}E_{--}^{++}C_{2}- \gamma_{+/}^{++}I_{+/}^{++}-I_{+/}^{++}\left( \omega+\psi^{++}\omega_{I} \right)+\alpha I_{+/}^{++}- \theta_{+-}I_{+/}^{++}+ \theta_{+/}I_{+-}^{++}$$

$$\frac{dI_{+-}^{++}}{dt}= \zeta^{++}E_{--}^{++}C_{3}- \gamma_{+-}^{++}I_{+-}^{++}-I_{+-}^{++}\left( \omega+{\psi^{++}\omega}_{I} \right)+ \alpha I_{+-}^{++}+\theta_{+-}I_{+/}^{++}- \theta_{+/}I_{+-}^{++}$$

$$\frac{dR_{--}^{++}}{dt}= \gamma_{++}^{++}I_{++}^{++}+ \gamma_{+/}^{++}I_{+/}^{++}+ \gamma_{+-}^{++}I_{+-}^{++}- {\omega R}_{--}^{++}- \rho^{++}R_{--}^{++}$$

We add the following equations, where “Q” is used to differentiate drug-resistant infectives from those with drug-sensitive strains, to capture emerging resistance for each immune category:

$$\frac{dQ^{--}}{dt}= {\phi_{++}^{--}}I_{++}^{--}+{\phi_{+-}^{--}}I_{+-}^{--}+{\phi_{+/}^{--}}I_{+/}^{--}$$

$$\frac{dQ^{+-}}{dt}= {\phi_{++}^{+-}}I_{++}^{+-}+{\phi_{+-}^{+-}}I_{+-}^{+-}+{\phi_{+/}^{--}}I_{+/}^{+-}$$

$$\frac{dQ^{++}}{dt}= {\phi_{++}^{++}}I_{++}^{++}+{\phi_{+-}^{++}}I_{+-}^{++}+{\phi_{+/}^{++}}I_{+/}^{++}$$

Relative Emergence Estimates

Determining the relative emergence attributable to each host category (represented by the symbol “$\phi$“ in the model) required estimates of the per cell, per bacterial generation mutation rate; the total number of infected cells per host; the expected number of bacterial generations per infection duration; the per category infection duration; and the relative success of the mutant strain. Whereas, in Chapter 1, these calculations were done during post-processing analysis for each antibiotic category, relative emergence is now computed within the ODE model, though the emergence remains record-keeping, since the emergent strain does not circulate independently.

**Table F. Relative Probability of Emergence**

As described in Appendix 1, each value for ϕ was computed using the per cell, per bacterial generation mutation rate; the total number of infected cells per host; the expected number of bacterial generations per infection duration; the per category infection duration; and the relative success of the mutant strain.

| **Symbol** | **Definition** | **Population** | **Value/Source** |
| --- | --- | --- | --- |
| $\phi_{++}^{--}$ | Relative probability of emergence in antibiotic-adherent, HIV/AIDS-host | Both | 1.12x10^-2[31, 32, 34]^ |
| $\phi_{+/}^{--}$ | Relative probability of emergence in partially adherent, HIV/AIDS-host | Both | 2.42 x10^-2^; assumed. |
| $\phi_{+-}^{--}$ | Relative probability of emergence in untreated, HIV/AIDS-host | Both | 2.23 x10^-3^; assumed. |
| $\phi_{++}^{+-}$ | Relative probability of emergence in antibiotic-adherent, HIV/AIDS+, HAART-, host | Both | 2.40 x10^-2^; assumed. |
| $\phi_{+/}^{+-}$ | Relative probability of emergence in partially adherent, HIV/AIDS+, HAART-,host | Both | 4.24x10^-2^; assumed. |
| $\phi_{+-}^{+-}$ | Relative probability of emergence in untreated, HIV/AIDS+, HAART-host | Both | 1.03x10^-2^; assumed. |
| $\phi_{++}^{++}$ | Relative probability of emergence in antibiotic-adherent, HIV/AIDS+, HAART+, hosts | Both | 1.12x10^-2^; assumed. |
| $\phi_{+/}^{++}$ | Relative probability of emergence in partially adherent, HIV/AIDS+, HAART+, hosts | Both | 2.42 x10^-2^; assumed. |
| $\phi_{+-}^{++}$ | Relative probability of emergence in untreated, HIV/AIDS+, HAART+, hosts | Both | 2.23 x10^-3^; assumed. |

**References**

1. DeNegre AA, Ndeffo Mbah ML, Myers K, Fefferman NH. Emergence of antibiotic resistance in immunocompromised host populations: A case study of emerging antibiotic resistant tuberculosis in AIDS patients. PLOS ONE. 2019;14(2):e0212969. doi: 10.1371/journal.pone.0212969.

2. CIA. Central Intelligence Agency World Factbook - Swaziland 2013. Available from: <https://www.cia.gov/library/publications/the-world-factbook/geos/wz.html>.

3. Todd J, Glynn JR, Marston M, Lutalo T, Biraro S, Mwita W, et al. Time from HIV seroconversion to death: a collaborative analysis of eight studies in six low and middle-income countries before highly active antiretroviral therapy. AIDS. 2007;21:S55-S63.

4. Deeks SG, Lewin SR, Havlir DV. The end of AIDS: HIV infection as a chronic disease. The Lancet. 382(9903):1525-33. doi: <http://dx.doi.org/10.1016/S0140-6736(13)61809-7>.

5. Organization WH. Global tuberculosis report 2012. Geneva: World Health Organization; 2012. Available from: h ttp://apps who int/iris/bitstream/10665/75938/1/9789241564502_eng pdf(Accessed 2013 June 6). 2012:66.

6. Steinbrook R. Tuberculosis and HIV in India. New England Journal of Medicine. 2007;356(12):1198-9. doi: doi:10.1056/NEJMp078049. PubMed PMID: 17377155.

7. Legido-Quigley H, Montgomery CM, Khan P, Atun R, Fakoya A, Getahun H, et al. Integrating tuberculosis and HIV services in low- and middle-income countries: a systematic review. Tropical Medicine & International Health. 2013;18(2):199-211. doi: 10.1111/tmi.12029.

8. Blower SM, Chou T. Modeling the emergence of the'hot zones': tuberculosis and the amplification dynamics of drug resistance. Nature Medicine. 2004;10(10):1111-6.

9. Trostle J. Inappropriate distribution of medicines by professionals in developing countries. Social Science & Medicine. 1996;42(8):1117-20. doi: <http://dx.doi.org/10.1016/0277-9536(95)00384-3>.

10. CIA. Central Intelligence Agency World Factbook - Indonesia 2013 Available from: <https://www.cia.gov/library/publications/the-world-factbook/geos/id.html>.

11. Morgan D, Mahe C, Mayanja B, Okongo JM, Lubega R, Whitworth JA. HIV-1 infection in rural Africa: is there a difference in median time to AIDS and survival compared with that in industrialized countries? AIDS. 2002;16(4):597-603.

12. Cohen T, Murray M. Modeling epidemics of multidrug-resistant M. tuberculosis of heterogeneous fitness. Nature Medicine. 2004;10(10):1117-21.

13. Dye C, Espinal MA. Will Tuberculosis Become Resistant to All Antibiotics? Proceedings: Biological Sciences. 2001;268(1462):45-52. doi: 10.2307/3067731.

14. Kaona F, Tuba M, Siziya S, Sikaona L. An assessment of factors contributing to treatment adherence and knowledge of TB transmission among patients on TB treatment. BMC Public Health. 2004;4(1):68. PubMed PMID: doi:10.1186/1471-2458-4-68.

15. Dye C, Williams BG. Criteria for the control of drug-resistant tuberculosis. Proceedings of the National Academy of Sciences. 2000;97(14):8180-5.

16. Tiemersma EW, van der Werf MJ, Borgdorff MW, Williams BG, Nagelkerke NJD. Natural History of Tuberculosis: Duration and Fatality of Untreated Pulmonary Tuberculosis in HIV Negative Patients: A Systematic Review. PLoS ONE. 2011;6(4):e17601. doi: 10.1371/journal.pone.0017601.

17. Corbett E.L. WCJWN, et al. The growing burden of tuberculosis: Global trends and interactions with the hiv epidemic. Archives of Internal Medicine. 2003;163(9):1009-21. doi: 10.1001/archinte.163.9.1009.

18. Mattapallil JJ, Douek DC, Hill B, Nishimura Y, Martin M, Roederer M. Massive infection and loss of memory CD4+ T cells in multiple tissues during acute SIV infection. Nature. 2005;434(7037):1093-7.

19. Mittler JE, Levin BR, Antia R. T-Cell Homeostasis, Competition, and Drift: AIDS as HIV-Accelerated Senescence of the Immune Repertoire. JAIDS Journal of Acquired Immune Deficiency Syndromes. 1996;12(3):233-48.

20. Helms T, Boehm BO, Asaad RJ, Trezza RP, Lehmann PV, Tary-Lehmann M. Direct Visualization of Cytokine-Producing Recall Antigen-Specific CD4 Memory T Cells in Healthy Individuals and HIV Patients. The Journal of Immunology. 2000;164(7):3723-32. doi: 10.4049/jimmunol.164.7.3723.

21. Nettle D. Why Are There Social Gradients in Preventative Health Behavior? A Perspective from Behavioral Ecology. PLoS ONE. 2010;5(10):e13371. doi: 10.1371/journal.pone.0013371.

22. Cheraghali A, Idries A. Availability, affordability, and prescribing pattern of medicines in Sudan. Pharm World Sci. 2009;31(2):209-15. doi: 10.1007/s11096-009-9282-3.

23. Kariuki S, Dougan G. Antibacterial resistance in sub-Saharan Africa: an underestimated emergency. Annals of the New York Academy of Sciences. 2014:n/a-n/a. doi: 10.1111/nyas.12380.

24. Kermack WO, McKendrick AG. A Contribution to the Mathematical Theory of Epidemics. Proceedings of the Royal Society of London Series A. 1927;115(772):700-21. doi: 10.1098/rspa.1927.0118.

25. McKendrick AG. Applications of Mathematics to Medical Problems. Proceedings of the Edinburgh Mathematical Society. 1925;44:98-130. doi: doi:10.1017/S0013091500034428.

26. Dye C, Scheele S, Dolin P, Pathania V, Raviglione MC, for the WHOGS, et al. Global burden of tuberculosis: Estimated incidence, prevalence, and mortality by country. JAMA. 1999;282(7):677-86. doi: 10.1001/jama.282.7.677.

27. Walley JD, Khan MA, Newell JN, Khan MH. Effectiveness of the direct observation component of DOTS for tuberculosis: a randomised controlled trial in Pakistan. The Lancet. 2001;357(9257):664-9. doi: <http://dx.doi.org/10.1016/S0140-6736(00)04129-5>.

28. Winnick S, Lucas DO, Hartman AL, Toll D. How Do You Improve Compliance? Pediatrics. 2005;115(6):e718-e24. doi: 10.1542/peds.2004-1133.

29. Aronson BS. Antibiotic-taking experiences of undergraduate college students. Journal of the American Academy of Nurse Practitioners. 2006;18(12):591-8. doi: 10.1111/j.1745-7599.2006.00184.x. PubMed PMID: 2009372802. Language: English. Entry Date: 20070309. Revision Date: 20120302. Publication Type: journal article.

30. Homedes N, Ugalde A. Patients' compliance with medical treatments in the third world. What do we know? Health Policy and Planning. 1993;8(4):291-314.

31. Billington O, McHugh T, Gillespie S. Physiological cost of rifampin resistance induced in vitro in Mycobacterium tuberculosis. Antimicrobial Agents and Chemotherapy. 1999;43(8):1866-9.

32. Dormans J, Burger M, Aguilar D, Hernandez-Pando R, Kremer K, Roholl P, et al. Correlation of virulence, lung pathology, bacterial load and delayed type hypersensitivity responses after infection with different Mycobacterium tuberculosis genotypes in a BALB/c mouse model. Clinical & Experimental Immunology. 2004;137(3):460-8. doi: 10.1111/j.1365-2249.2004.02551.x.

33. Stone KC, Mercer RR, Gehr P, Stockstill B, Crapo JD. Allometric Relationships of Cell Numbers and Size in the Mammalian Lung. American Journal of Respiratory Cell and Molecular Biology. 1992;6(2):235-43. doi: 10.1165/ajrcmb/6.2.235.

34. Gill WP, Harik NS, Whiddon MR, Liao RP, Mittler JE, Sherman DR. A replication clock for Mycobacterium tuberculosis. Nat Med. 2009;15(2):211-4. doi: <http://www.nature.com/nm/journal/v15/n2/suppinfo/nm.1915_S1.html>.

35. Tam VH, Louie A, Deziel MR, Liu W, Leary R, Drusano GL. Bacterial-Population Responses to Drug-Selective Pressure: Examination of Garenoxacin’s Effect on Pseudomonas aeruginosa. Journal of Infectious Diseases. 2005;192(3):420-8. doi: 10.1086/430611.

36. Willing BP, Russell SL, Finlay BB. Shifting the balance: antibiotic effects on host–microbiota mutualism. Nature Reviews Microbiology. 2011;9(4):233-43.

37. Baquero F, Negri M-C, Morosini M-I, Blázquez J. Antibiotic-selective environments. Clinical Infectious Diseases. 1998;27(Supplement 1):S5-S11.

38. Kolář M, Urbánek K, Látal T. Antibiotic selective pressure and development of bacterial resistance. International Journal of Antimicrobial Agents. 2001;17(5):357-63. doi: <http://dx.doi.org/10.1016/S0924-8579(01)00317-X>.

39. Brunham RC, Plummer FA, Stephens RS. BACTERIAL ANTIGENIC VARIATION, HOST IMMUNE-RESPONSE, AND PATHOGEN-HOST COEVOLUTION. Infect Immun. 1993;61(6):2273-6. PubMed PMID: WOS:A1993LE49800001.

40. Blaser MJ, Kirschner D. The equilibria that allow bacterial persistence in human hosts. Nature. 2007;449(7164):843-9. doi: <http://www.nature.com/nature/journal/v449/n7164/suppinfo/nature06198_S1.html>.

41. Rubin RH. Fungal and bacterial infections in the immunocompromised host. Eur J Clin Microbiol Infect Dis. 1993;12(1):42-S8. doi: 10.1007/bf02389877.

42. Anderson KG. Life expectancy and the timing of life history events in developing countries. Human Nature. 2010;21(2):103-23.

43. Quinn TC. HIV epidemiology and the effects of antiviral therapy on long-term consequences. AIDS (London, England). 2008;22(Suppl 3):S7.

44. Chan MF. The Impact of Health Care Resources, Socioeconomic Status, and Demographics on Life Expectancy: A Cross-Country Study in Three Southeast Asian Countries. Asia-Pacific Journal of Public Health. 2013. doi: 10.1177/1010539513475650.

45. Hull TH, Hull VJ. Population Change in Indonesia: Findings of the 1980 Census. Bulletin of Indonesian Economic Studies. 1984;20(3):95-119. doi: 10.1080/00074918412331334712.
